# Old Practice, but Young Research Field: A Systematic Bibliographic Review of Personal Branding

**DOI:** 10.3389/fpsyg.2020.01809

**Published:** 2020-08-11

**Authors:** Stefan Scheidt, Carsten Gelhard, Jörg Henseler

**Affiliations:** ^1^Chair of Product-Market Relations, Department of Design, Production and Management, University of Twente, Enschede, Netherlands; ^2^Nova Information Management School, Universidade Nova de Lisboa, Lisbon, Portugal

**Keywords:** personal branding, personal brand, human brand, literature review, bibliographical analysis

## Abstract

Before engaging with the already intensive and still increasing personal branding activities in many fields of practice, a scholarly approach would call for a more specific definition of the concept of personal branding processes and the resulting human brands. A multi-step analysis of the growing body of literature on personal branding is employed, integrating a framework that covers six key research streams of personal branding, (1) terminology and definition, (2) underlying theories, (3) classes and categories, (4) benefits, (5) antecedents, and (6) key ingredients and applications, complemented by challenges the domain of personal branding has to cope with. The analysis shows that personal branding is an interdisciplinary concern, which is still in its infancy and in which universally valid personal branding frameworks or even theories cannot be identified yet. Personal branding appears as a source for new academic impulses, as it may sensitize scholars to opportunities for intensifying collaboration with practitioners and with other academic domains to enrich and disseminate knowledge in their fields.

## Introduction

While “personal branding” and “human brands” as terms are a modern invention, the branding of individuals is as old as human interaction and society itself. Alexander the Great has been claimed as the first celebrity in human history (Braudy, 1997), the archetypal erudite Goethe achieved success by using all elements of the marketing mix in order to differentiate himself from other authors of his time (Bendisch et al., 2013), and Andy Warhol's “idea that ‘everyone will be famous for 15 min’ comments on a world where image reigns supreme” (Schroeder, 2005, p. 1294). Personal branding has become a vital part of individuals, society, culture, and economy. Research has responded accordingly, with different academic disciplines converging on this subject over time, each focusing on many different aspects. Goffman (1956) described self-presentation as the intentional and tangible component of identity, human brands have been a defining characteristic of the broadening of the traditional concept of marketing (Kotler and Levy, 1969), and, from a social psychology perspective, rarity and stability affect celebrity authenticity (Moulard et al., 2015) to name just a few developments in the field.

Whereas personal branding as a term is a relatively recent invention, the reality behind it is not. The significant increase of scientific attention to personal branding, especially in the last 10 years, has given this contemporary phenomenon widespread, albeit fragmentary academic presence. Schau and Gilly (2003), investigating self-presentation in the Web 1.0 environment, and Thomson (2006), exploring why consumers form strong attachments to human brands, published the first scientific articles to empirically examine human brands. More and more empirical studies have been conducted in the field in the last few years (e.g., Parmentier et al., 2013; Hofmann et al., in press), but they remain few and far between. Several personal branding frameworks have also been put forward, some based on these empirical studies (e.g., Khedher, 2019) and some on more conceptual work (e.g., Bendisch et al., 2013), but a comprehensive personal branding framework, let alone a sustainable theory, is yet sorely missing for academic purposes. Moreover, the key question as to whether science can “reclaim self-marketing and personal branding from the enthusiasts” (Shepherd, 2005, p. 12) is still waiting for an academically valid answer.

As research is continually developing both in terms of breadth, going into new directions, e.g., studying bloggers and influencers in social media, and depth, with more studies covering well-known topics, such as brand attributes, it seems an opportune moment for an updated review of current literature. To address the mentioned lacuna, the objective of this paper tries to present an analysis of the growing body of literature on personal branding, covering its terminology and definitions, underlying theories, classes and categories, benefits, antecedents, key ingredients and applications as well as its challenges. In doing so, this review contributes significantly to the positioning of personal branding in the applied psychology, branding, and business research context by bundling fragmented ideas and structuring single key aspects. Such an approach complements to the interdisciplinary review on personal branding by Gorbatov et al. (2018), systematically links the underlying and existing body of knowledge and opens avenues for future novel research (Palmatier et al., 2018).

## Methodology

The fragmented and not clearly arranged field of knowledge in personal branding does not benefit from providing merely a more comprehensive overview on existing literature. Rather, we aim to identify trends and key research streams in personal branding resulting in constructive criticism of existing work and avenues for future research. Therefore, a structured approach that implies a bibliographic analysis in its core is suited for the method of choice (Paul and Singh, 2017; Ferreira, 2018).

**Figure 1:**
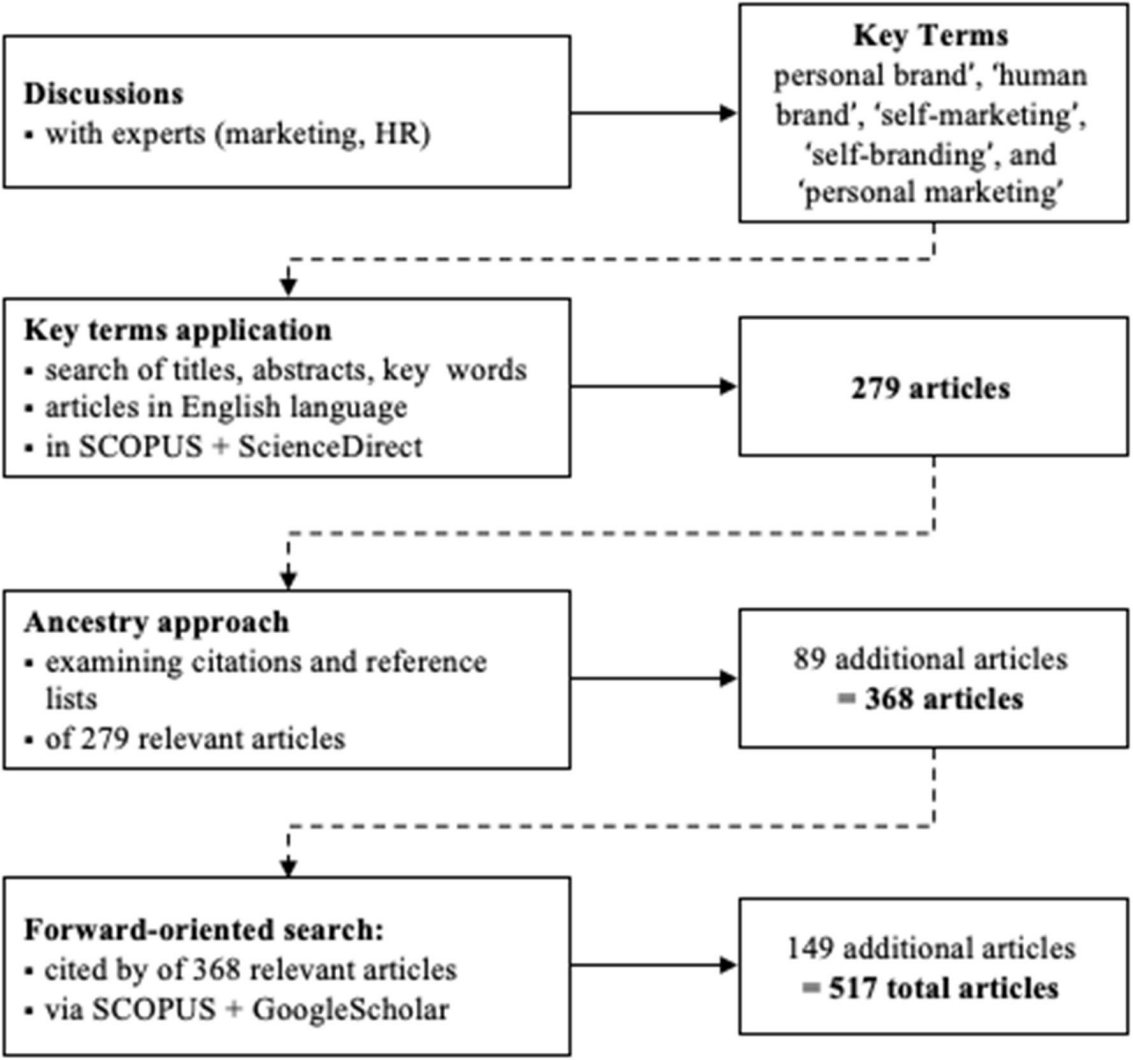
Review methodology process.

The current body of literature on the subject to be studied has been surveyed systematically (works published before 31 December 2019), complementing this bibliographical data with substance-centered research in a loop of cross-fertilization that enriched both perspectives (Figure 1).

During pre-analysis a pilot study was conducted in which chosen experts, such as top managers responsible for corporate HR, HR managers doing recruitment and development at managerial level, and professionals who deliver communication services for CEOs and other branded individuals were invited to join several discussions (see Table 1). This first step was used initially to improve awareness of previous research into the branding of individuals. It revealed the following set of key terms for further enquiry: “personal brand,” “human brand,” “self-marketing,” “self-branding,” and “personal marketing.”

**Table 1:** The expert sample group.

**Interviewees**	**Age**	**Gender**	**Nationality**	**Function**	**Educational background**	**Industry**
Expert 1	51	Female	German	CEO	Business marketing, architecture	Real estate/Construction
Expert 2	57	Male	Austrian	SVP Corporate Communications, Corporate Marketing	Philosophy, literature	Utilities
Expert 3	48	Female	German	HR Manager	Business economics (industrial psychology)	Utilities
Expert 4	43	Male	German	Managing Director	English literature, political science, Anglo-American history	Advertising
Expert 5	50	Male	German	CEO	Banker, business economics (marketing)	Advertising
Expert 6	39	Female	German	HR Director	Business economics (marketing and labor)	Mobility
Expert 7	46	Male	Spain	HR Manager	Business economics	Automobile

For the purposes of the main analysis, the chosen terms were applied in a search of the titles, abstracts, and keywords of articles in the English language. This analysis required good coverage of branding- and business-related research in multiple disciplines, for which SCOPUS and ScienceDirect are suitable and popular databases.

**Figure 2:**
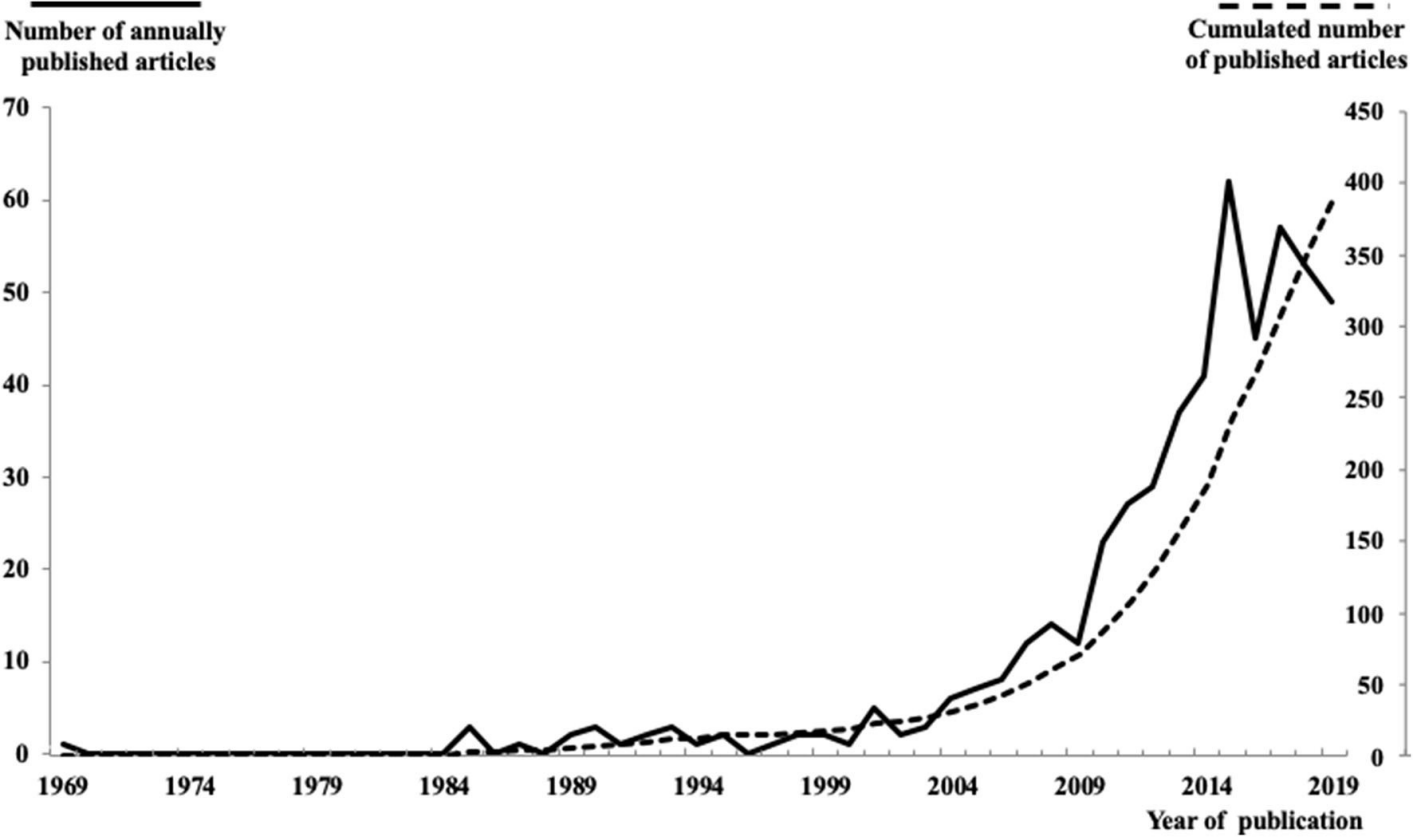
Publication of academic articles with relevance to personal branding over time.

Following this first search and to ensure the most exhaustive literature review possible, the ancestry approach (Cooper, 1989, 2010; Atkinson et al., 2015) was applied to identify additional articles. This backward search uncovers new articles of interest that meet the criteria by examining the citations and the reference list of the articles already available to the researchers (e.g., Cornwell and Maignan, 1998; Xyrichis and Lowton, 2008; Filo et al., 2015). To compensate for the main limitation of the ancestry approach, i.e., its one-sided retrospective direction, citation research (cited by) via SCOPUS and GoogleScholar was conducted as a forward search on all academic articles to cover relevant later citations. In addition, articles published after 2019, but before the manuscript was finalized were added to the review. In total, 518 articles were included in this review whose publication started in 1969 and increased significantly after the mid-2000s (Figure 2).

The following subject matter research analyzed and matched the articles with each other via a fine-grained and critical reading to support both an immediate check of the thematic relevance of the articles and identification of the key topics in personal branding.

## Findings

Six key research streams were identified and served as a framework to investigate the chosen articles with a view to relevant key topics (Table 2). (1) It opens by reviewing what a human brand and personal branding respectively are, proposing updated terminologies and definitions; (2) This leads to a consideration of the main psychological theories in their application to personal branding; (3) Followed by an examination of the classes and categories in which personal branding is applied by highlighting the difference between celebrities, icons, and branded ordinary people; (4) The benefits of personal branding reveal its importance for the branded individual as well as for stakeholders; (5) It then focuses on the antecedents of the contemporary phenomenon of personal branding, answering the question of the reasons for which it has been spreading; (6) Key ingredients and applications indicate how human brands emerge and how it is applied in a branding context and beyond. Subsequently, the challenges that appear with personal branding have been elaborated, as a critical view on personal branding is needed to support a responsible and conceptually meaningful development of future concepts and theories.

**Table 2:** The structure of scholarly knowledge on personal branding: research streams, concepts, and contributions.

**Research streams**	**Concepts**	**Contributions**
Terminology	Personal branding	Lair et al., 2005; Shepherd, 2005; Harris and Rae, 2011; Labrecque et al., 2011; Manai and Holmlund, 2015; Dumont and Ots, 2020
	Human branding	Thomson, 2006; Close et al., 2011; Carlson and Donavan, 2013
	Self-branding	Hearn, 2008; Gandini, 2016; Duffy and Pooley, 2019
	Self-marketing	Shepherd, 2005; Shuker, 2014
	Corporate personhood	Gershon, 2014
Definition	Individual contribution	McNally and Speak, 2002; Clark, 2011; Morton, 2012; Gander, 2014
	Focus on the audience	Parmentier et al., 2013; Philbrick and Cleveland, 2015; Preece and Kerrigan, 2015
	Differentiation	Parmentier et al., 2013; Gander, 2014; Lunardo et al., 2015
	Focus on career	Lair et al., 2005; Shepherd, 2005; Morton, 2012; Gershon, 2014; Gorbatov et al., 2019
	Commercialization	Fillis, 2015; Preece and Kerrigan, 2015
Theories	Attachment theory	Thomson, 2006; Huang et al., 2015; Loroz and Braig, 2015; Saboo et al., 2016
	Self-determination theory	Ryan and Deci, 2000; Huang et al., 2015 Moulard et al., 2015
	Attribution theory	Moulard et al., 2015
	Social identity theory	Carlson et al., 2009; Carlson and Donavan, 2013
	Cue utilization theory	Close et al., 2011; Zamudio et al., 2013; Moulard et al., 2014
	Structuration theory	Turner, 2004; Lindridge and Eagar, 2015
Benefits	Career	Close et al., 2011; Harris and Rae, 2011; Parmentier and Fischer, 2012; Zamudio et al., 2013; Moulard et al., 2015; Philbrick and Cleveland, 2015; Gorbatov et al., 2019
	Differentiation	Shepherd, 2005; Chen, 2013
	Improvement of the self	Hearn, 2008; Gall, 2010; Gander, 2014; Philbrick and Cleveland, 2015; Preece and Kerrigan, 2015
	Visibility and attention	Thomson, 2006; Hearn, 2008; Harris and Rae, 2011; Fillis, 2015; Philbrick and Cleveland, 2015
	Sales/risk reduction	Schroeder, 2005; Carlson and Donavan, 2013; Moulard et al., 2014; Huang et al., 2015; Mills et al., 2015; Preece and Kerrigan, 2015
	Identity construction	Thomson, 2006; Cocker et al., 2015; Huang et al., 2015; Lunardo et al., 2015
	Co-branding effects	Close et al., 2011; Bendisch et al., 2013; Moulard et al., 2014; Zinko and Rubin, 2015; Scheidt et al., 2018
	Impact on society	Schroeder, 2005; Fillis, 2015; Lindridge and Eagar, 2015
Antecedents	New world of work	Lair et al., 2005; Hearn, 2008; Gershon, 2014; Philbrick and Cleveland, 2015; Vallas and Cummins, 2015; van Oort, 2015; Gandini, 2016
	Development of media	Schau and Gilly, 2003; Lair et al., 2005; Hearn, 2008; Dutta, 2010; Gehl, 2011; Labrecque et al., 2011; Chen, 2013; Elwell, 2014; Fillis, 2015; Mills et al., 2015; Gandini, 2016
	New individualism	Lair et al., 2005; Hearn, 2008; Gershon, 2014; Lindridge and Eagar, 2015
	Existential angst	Lair et al., 2005; Shepherd, 2005; Harris and Rae, 2011; Labrecque et al., 2011
Key ingredients and applications	Personality	Chen, 2013; Arai et al., 2014; Fillis, 2015; Philbrick and Cleveland, 2015
	Authenticity	Thomson, 2006; Morton, 2012; Gander, 2014; Moulard et al., 2014, 2015; Lunardo et al., 2015; Mills et al., 2015; Philbrick and Cleveland, 2015; Kowalczyk and Pounders, 2016
	Differentiation	Labrecque et al., 2011; Parmentier and Fischer, 2012; Carlson and Donavan, 2013; Chen, 2013; Gander, 2014; Cocker et al., 2015; Mills et al., 2015
	Visibility	Harris and Rae, 2011; Labrecque et al., 2011; Morton, 2012; Parmentier and Fischer, 2012; Elwell, 2014; Gander, 2014; Philbrick and Cleveland, 2015
	Social media	Hearn, 2008; Gehl, 2011; Labrecque et al., 2011; Chen, 2013; Elwell, 2014; Lindridge and Eagar, 2015; Gandini, 2016
	Narrative identity	McAdams, 2011; Belk, 2013; Elwell, 2014
	Attachments	Thomson, 2006; Carlson and Donavan, 2013; Chen, 2013; Loroz and Braig, 2015
	Co-brands and stakeholders	Close et al., 2011; Parmentier and Fischer, 2012; Bendisch et al., 2013; Parmentier et al., 2013; Preece and Kerrigan, 2015; Speed et al., 2015; Dumont and Ots, 2020
	Brand equity	Hearn, 2008; Parmentier and Fischer, 2012; Moulard et al., 2014; Chen and Chung, 2016; Cottan-Nir, 2019

### What Is a Human Brand? What Is Personal Branding?

#### Terminology

The branding of individuals has introduced a diverse set of new terms into the jargon of professionals and academics alike. Brand You (Peters, 1999), Brand Yourself (Andrusia and Haskins, 2000), and me Inc (Peters, 1999) are neologisms introduced by marketers and professionals who focus primarily on a person-centered approach “constructing a product based on themselves that can then be marketed as effectively as possible” (Shepherd, 2005, p. 6). At the same time, academic efforts in this field suffer from a lack of consistent terminology, with terms such as self-branding, self-marketing, or corporate personhood. However, personal branding and, to a limited extent, human branding remain the most frequently used and accepted terms. “Personal branding” was coined in 1997 by Tom Peters in the Fast Company Magazine. Although Peters did not give an explanation or definition, “the phrase ‘personal branding’ is now fairly well established, and more consistently used” (Shepherd, 2005, p. 2), which can be confirmed by the research produced for this literature review.

#### Definitions

Personal branding and its various synonyms are frequently used without any explicit or even consistent definitions of the term, giving rise to a certain degree of terminological fuzziness. A deeper investigation of the definitions identifies, nonetheless, some common ground and suggests a conceptual approach based on three pillars: First, what the branded individual brings in. This implies personal qualifications, such as skills, competencies, experience, or expertise. Second, a focus on the audience or specific target groups and their perceptions of the branded person and relevant associations. This aspect ranges from few specific definitions, such as “the employee trying to impress his boss” (Kotler and Levy, 1969, p. 12) and psychologists' “clients” (Cederberg, 2017) to mostly very abstract circumscriptions like “who you are to the world around you” (Philbrick and Cleveland, 2015, p. 183) or “to a target audience” (Parmentier et al., 2013, p. 382). Third, differentiation appears as the end product, and the process of personal branding aims to produce a distinction from peers by leveraging one's points of difference and defining individual unique selling proposition. Beside this conceptual triangle, a strong focus on career and employment as well as the commercialization of the branded individual can be identified as other particularly key aspects.

A distinction between the process, i.e., personal branding, and the thing, i.e., the human brand, is obvious and can traced back to the differing philosophies of Heraclitus and Democritus (Rescher, 1996). However, a large majority of the reviewed articles do not distinguish clearly between these two perspectives. In this vein, confusion arises as “personal branding” serves as a term for the entire phenomenon of the branding of individuals, whereas “personal brand” is used to describe the outcome of personal branding in general as well as the class of ordinary people and field-specific individuals who do not own a celebrity status.

On the whole, updated definitions for the contemporary phenomenon of personal branding and its statement as a resulting brand need to be determined. Therefore, the following definitions can be proposed: “Personal branding” could remain as a term from a process perspective and, thus, *is the entire process of establishing, maintaining, and developing an individual's human brand*. It includes the active and selective integration of certain personal preconditions with due consideration for the changing nature of the field in which the human brand is to be established. In order to resolve terminological confusion, “human brand” could serve as a general term for the brand that results from a personal branding process, independent of class. In this sense, a human brand *is an intangible asset linked to a person, which generates economic and social value through its visibility as a result of a personal branding process*. Whereas, the personal branding process is a collective act between stakeholders and the branded individual's entire personality, visibility is expected to arise within and beyond her/his professional field to stand apart from other human brands and to fit into a defined target market. All aspects of the definitions proposed before will be considered during the course of this literature review.

### Underlying Theories

Not surprisingly, scholars refer to theories and concepts from the discipline of branding in their understanding of personal branding, such as Keller's (1993) customer-based brand equity model that serves as a basis for the concept of athletes' brand images (Arai et al., 2014) and the idea of human brand equity for football players (Parmentier and Fischer, 2012) or the model of establishing points of differentiation and points of parity (Keller et al., 2002). However, the interdisciplinary domain of personal branding does not only call for a broader approach in underlying theories and concepts. Rather, theories specifically from psychology are used to do justice to the components “personal” or “human” in personal branding and human brand.

#### Attachment Theory

This psychological, evolutionary and ethological theory (Bowlby, 1969) serves as a foundation for a detailed investigation into personal branding, as “understanding how to create or intensify attachments could offer both an effective and an economical means of achieving stronger marketing relationships that may prevent consumer defections, increase consumers' forgiveness in the face of negative information, and can predict brand loyalty and willingness to pay” (Thomson, 2006, p. 105). Whereas, autonomy, relatedness, and competence serve as antecedents of the strength of people's attachment to human brands, Loroz and Braig (2015) create an empirically more comprehensive and sophisticated picture of consumer attachments to human brands. Thus, the importance of the competence dimension to develop strong human brand attachment depends on the extent to which the human brand maintains competence. In addition, brand appeal, consistent focus, and longevity are effective moderators of human brand attachment strength and dimensions such as favorability, originality, and clarity should be included for a broader understanding of human brands.

#### Self-Determination Theory

Regarding the perception of a celebrity's authenticity (Moulard et al., 2015), self-determination theory in its focus on human motivation and personality proposes that intrinsically motivated behavior is “authentic in the fullest sense of those terms” (Ryan and Deci, 2000, p. 74). Intrinsic motivation in turn is characterized by participation in an activity for its inherent satisfaction of three innate psychological needs that are essential for optimal functioning: autonomy (i.e., need to perceive origin of source of one's own behavior), relatedness (i.e., need to feel connected with others), and competence (i.e., need to have an effect on one's outcomes and surroundings). Autonomy, relatedness, and competence are therefore assumed to be prerequisites for the authenticity of celebrities.

#### Attribution Theory

Derived from attribution theory, rarity and stability are suggested as the main components of human brands (Moulard et al., 2015) as they are expected to contribute to authenticity. The augmentation principle within the attribution theory is used to derive rarity, since it states that that actions that involve costs, risks, or sacrifices (i.e., nonconforming behaviors) are more likely to be attributed to the person than to external causes. This hypothesis is grounded in the idea that it is often difficult to express one's true self, with social pressure causing most people to adapt to the norm. Therefore, it is difficult to “go against the grain” because social acceptance is less likely to be achieved. People who do so are more likely to be perceived as intrinsically motivated. This idea is in line with previous research (Anton, 2001; Vannini and Franzese, 2008) indicating that social conformity and impression management are the antithesis of authenticity. Stability, in turn, is justified on the grounds that similar behavior in various situations and similar behavior in response to distinct stimuli/units may collapse to similar behavior over time. Thus, consumers' perceptions of a celebrity's authenticity are driven by the fact that that the behavior is unique to that person and is stable over time. Concerning the age of the celebrities' target group, younger people are more likely to rely on rarity than older people when judging the authenticity of celebrities, while older people rely primarily on stability when assessing the authenticity of a celebrity. Consequently, a celebrity's authenticity is influenced by the rarity and stability antecedents, yet the relative weights of these antecedents evolve with age.

#### Social Identity Theory

According to this theory originally formulated by social psychologists, consumers demonstrate membership in a particular social category by associating themselves with a personal brand, thus creating a social identity. Carlson and Donavan (2013) investigated the extent to which brand personality attributes of professional athletes influence consumer-brand relationships with a professional sports team. They used social identity theory as a framework for a model that predicts consumer connections with athletes and the team, retail spending and number of games watched. According to social identity theory, self-categorization into a group serves a self-definitional role that helps individuals make sense of the world (Tajfel and Turner, 1985; Hogg et al., 1995). Social identification serves as a source of self-esteem that should be enhanced by membership in a valued group. Here, strong identification with the group should go hand in hand with positive evaluation of the ingroup (Leary and Tangney, 2012). Consequently, consumers are drawn to sports teams that have a strong “similarity” to their own actual or ideal self (Madrigal and Chen, 2008; Carlson et al., 2009; Fink et al., 2009). Carlson and Donavan (2013) suggest that likewise, consumers should be drawn to individual athletes perceived to be similar to their own actual or ideal self. They identify with famous athletes because they are perceived to be symbolic of desirable reference groups and being associated with the athlete's brand personality attributes may enhance their own self-image. Additionally, consumers are more likely to identify with a player who is perceived to be both prestigious and distinctive. These findings are in line with social identity theory, which suggests people seek to differentiate themselves from others in social contexts and are thus likely to affiliate with entities that enhance their self-esteem (Tajfel and Turner, 1985; Leary and Tangney, 2012). In contrast to more traditional brands, human brands have the unique opportunity to successfully differentiate themselves from the consumer's perspective and to offer social identification even through negative characteristics. The image of being rebellious is often perceived as being highly desirable since, for instance, many celebrities and athletes are very popular among consumers because of their negative “bad boy” or “bad girl” images (Burton et al., 2001).

#### Cue Utilization Theory

An application of cue utilization theory enables to differ between intrinsic and extrinsic cues of human brands which is comparable to Keller's (1993) distinction between product-related, i.e., a product's physical composition, and non-product-related brand attributes, e.g., price and packaging. Investigating artist brands from the point of view of cue utilization theory (Moulard et al., 2014) the appearance and the quality of the artwork itself can be conceptualized as an intrinsic cue whereas the attitude toward the artist, or the artist's brand equity, can be conceptualized as an extrinsic cue. Doctoral candidates' brand attributes are categorized into intrinsic and extrinsic cues, each with a positive impact on certain aspects of the candidates' job search process (Close et al., 2011). Whereas, the candidates' research productivity and dissertation progress are attributed to the intrinsic cues, the extrinsic cues are represented by granting faculty research productivity, advisor research productivity, and doctoral consortium attendance. Additionally, doctoral candidates' publications in top ranked journals strengthen the confidence that a candidate's publication in a particular journal meets a certain quality standard and thus served as important predictors of the candidate's placement success. This is consistent with the cue utilization theory, suggesting that some cues have higher predictive and confidence values than other cues (Olson, 1977; Richardson et al., 1994), and that cues with such high values are given the greatest weight in assessing quality. The predictive value of a cue is directly connected to the degree to which the evaluators associate the cue with quality.

#### Structuration Theory

This theory explains how social systems are created and reproduced through the engagement of structure and individual's, group's or organization's behavior (Giddens, 1984) and is utilized to explore the extent that celebrities' human brand can emancipate themselves from a character they are associated with (Lindridge and Eagar, 2015). Exemplarily applied to the late singer, songwriter and actor David Bowie, the structure of his human brand can be understood through the interactions and knowledge between so-called “agents,” i.e., producers, managers, agents, publicists and the entire machinery of the music industry, who work with and sometimes even force the artists to construct and perform their persona. Consequently agents' behaviors are not only determined by the structure that they exist within but are also constantly recreated and adapted through differing time periods. Emphasizing the question about the ownership of a human brand and its characters leads to the recognition of celebrities as image-creators and -prisoners depending on which agents hold the power to influence image associations. In this respect, structuring theory is expected to enable scholars to deal with this conflict by considering how agents within the structure can influence the agency of a human brand, leading to an “ongoing negotiation between the different agents within the celebrity structure” (Turner, 2004).

### Classes and Categories

Regarding the fact that scholars primarily use the term “personal brand” when considering or investigating ordinary people as brands, three different classes of human brands are proposed: the celebrity, the personal brand, and the icon.

#### Classes

Celebrities, defined as “part of the social elite who engage in the public relations machine of television and movie roles, special event appearances and talk show and gossip magazine placements” (Lunardo et al., 2015, p. 687), enjoy great popularity in personal branding among both practitioners and scholars. While the very first empirical studies of personal branding targeted celebrities (Thomson, 2006), recent investigations have evolved to form a separate interdisciplinary research area beyond a personal branding perspective, particularly in the last few years, as expressed in its publication platform in the *Celebrity Studies* journal. Nonetheless, the search through key terms in this review resulted in 83 articles that focus on celebrities confirming the manifest interest of scholars in investigating them.

The chronological aspect of the demographics of celebrity culture comes into play when turning to the branding of ordinary people. Because of the contradictory forces affecting media visibility, namely the need for constant renewal and the competition for that scarce resource that is public attention, there is a rapid turnover of celebrities in the media. Whereas, traditional media, such as television, radio, and newspapers, had been the exclusive domain of corporate entities and celebrities, social media allows all individuals to create their own unique virtual spaces and to reach broader audiences irrespective of time or place. How much room is there for celebrities to not fall out from the celebrity zone and step into the zone of ordinary human brands, i.e., personal brands? In turn, micro-celebrities appear as an intermediate stage during the transition from a personal brand to celebrity status (Khamis et al., 2017) enabled by social media.

Finally, the icon is a legitimate cultural symbol of personal achievement and societal values. While celebrities, for a period of time, own the symbolic meanings associated with their private and public selves, icons experience a convergence and transformation of meanings across time, reflecting wider cultural concerns. Celebrities transition into icons when their fame endures through the transformation of their cultural meaning and values that mirror changes in society (Lindridge and Eagar, 2015). Even if terms such as “superstar” and “idol” (Epstein, 2005) may confirm that a consistent separation does not exist, an icon stands out from the crowd of an increasing number of celebrities.

Scholars tend to prioritize some areas in their research, while others still lack scholarly attention (Table 3) regarding the assignment of three human brand classes to 11 different categories (see Appendix 1).

**Table 3:** Number of publications focusing on three different classes of human brands in eleven different categories.

**Categories**	**Sub-categories**	**Classes of human brands**		
		**Icon**	**Celebrity**	**Personal brand**
Sports	Athletes	0	4	30
	Athletic trainers	0	0	2
	Athletes from specific disciplines	2	7	6
Academics	General	0	0	9
	Professors	0	0	4
	Students	0	0	22
Politicians	General	0	2	8
	Prime ministers	0	4	0
	Presidents	0	4	0
	Election candidates	0	1	4
	Ordinary politicians	0	0	1
Visual artists	General	0	0	7
	Painters	0	4	0
	Sculptors	0	1	0
	Video/film producers	0	1	0
Performing artists	Actors	0	6	1
	Musicians	2	4	6
	Comedians	0	1	0
	Models	0	4	2
	TV Anchor	0	2	1
Aristocracy	Royals	1	0	1
Producers of hedonic products	Chefs	0	3	0
Professional services	General	0	0	6
	Medical staff	0	0	10
	Consultants	0	0	1
	IT professionals	0	0	1
	Engineers	0	0	2
	Salespeople	0	0	2
	Teachers	0	0	1
	Librarians	0	0	6
Self-employed	General	0	0	1
	Creative industry	0	0	3
	Spiritual	0	0	1
Business managers	CEOs	0	0	15
	Executives	0	0	2
	Leaders	0	0	3
	Entrepreneurs	0	2	5
	Owner-manager	0	0	1
Content creators	Journalists	0	0	22
	Authors	0	1	3
	Influencers	0	1	1
	Bloggers	0	0	16
	YouTubers	0	1	5
	Vloggers	0	2	2

#### Categories

By reason of the increasing presence in the public and in the media, *celebrities* are no longer limited to actors and musicians. It covers athletes, business persons, technology entrepreneurs, politicians, scientists, and others as well (Moulard et al., 2015). Celebrities are primarily studied in the sports category, such as Andy Murray, and in politics, for example Kevin Rudd and Donald Trump. However, performing artists, such as Oprah Winfrey, represent the largest category of celebrities being investigated. Whereas, these three categories mirror the high level of attention they have in the media, royals or the family members of higher or lower aristocracy have rarely yet been targeted by scholarly interest. In a similar vein, visual artists, business managers, and content creators have been the subject of only few studies of celebrities, despite their intense visibility in different kinds of media.

*Personal brands* in the category of content creators have been facing increasing scholarly interest in the last few years, especially journalists, bloggers, and YouTubers. Similarly, professors and students face intense academic attention, which might simply be caused by the ready availability of the sample group. Whereas, personal brands in the sports category emphasize the athlete in general, numerous different jobs have been studied in the professional services, such as doctors and nurses in the medical field, psychologists, librarians, and engineers. Business managers, for example CEOs, represent another category studied more frequently. However, the personal brands in the categories of the producers of hedonic products and the aristocracy suffer from a lack of scholarly interest.

The number of branded individuals being labeled and studied as *icons* is very low so far, which mirrors their hardly existing availability for research efforts. British royals as human brands, for example, serve as “iconic British symbols” (Otnes and Maclaran, 2018, p. 9). Ziggy Stardust, a character derived from the performance persona of David Bowie, represents “a powerful icon of a time, place, ethos and subculture that would never age and is infinitely replicable and symbolically malleable, in ways that the celebrity cannot reproduce” (Lindridge and Eagar, 2015, p. 24). David Beckham, who embraces multiple masculinities, including the romantic and compassionate husband, the hands-on father, the football legend, and the fashionable style icon (Cocker et al., 2015), mirrors a societal shift in positioning himself as a metrosexual human (Parmentier and Fischer, 2012).

Nonetheless, the category of aristocracy in general is still an under-investigated area that calls for deeper investigation, similar to some others. Although Kotler and Levy (1969, p.10) already stated that “political contests remind us that candidates are marketed as well as soap,” and despite the fact that political marketing is big business, particularly in the United States, and that it attracts sophisticated investigation from scholars (e.g., Butler and Harris, 2009; Hughes and Dann, 2009; Algara, 2019), the personal branding of politicians still lacks empiric consideration (Harris and Rae, 2011; Gershon, 2014). In same vein, the art market offers numerous well-known brands of visual artists, like Dali, Picasso, van Gogh, Rembrandt, or Warhol, all of whom are imbued with celebrity status (Schroeder, 2005; Fillis, 2015).

### Benefits of Personal Branding

First and foremost, the branded individual benefits generally from personal branding when competing for work, seeking advancement in specific occupations or professions, or pursuing a career path that leads to higher financial earnings. This fundamental advantage includes other specific aspects, such as lucrative endorsement opportunities for athletes, the self-branded business ventures of celebrities, or entry-level placement as well as entry salary premiums in the marketing job market. While these benefits are closely linked to differentiation as a consequence of personal branding and can be traced back to the idea of a new world of work, personal branding is thought to offer some impact to the individual personality as well. Continuous self-reflection throughout the personal branding process may lead to continuous learning and thus enable the branded individuals to improve their abilities and achieve much greater self-awareness, self-esteem, self-confidentiality, and self-worth. Visibility acts as a beneficial consequence of personal branding too, enhancing social capital for the branded individual.

Turning to the target groups of personal branding, human brands as endorsers are a major force driving retail sales when products are associated with them. In the art market in particular, personal branding serves as a means for reducing risks and increasing the willingness to pay a premium price. Beside commercial considerations, psychological aspects are of interest, such as consumer-human brand attachment that may advance identity construction. Celebrities complement or even replace the family system for identity construction of young people. The family system and the family subsystem are interconnected to satisfy the basic human needs of belonging, autonomy and distinctiveness, all of which are essential for identity development (Scabini and Manzi, 2011). The fulfillment of psychological needs, such as autonomy, relatedness, or competence as well as appearance attractiveness seems to be of great importance in the transition from parental attachment to idol attachment for young people especially. Identity includes, but is not limited to gender, race, ethnicity, spirituality, sexuality, and social class (Dillon et al., 2011). The latter has not been at the forefront of the marketing literature on celebrity but is an important part of the appeal of many celebrity brands and, thus, a crucial factor for identity construction at consumer side. For instance, several working-class celebrities based in Britain, such as Kerry Katona, Jade Goody and Wayne Rooney, have labeled themselves ‘chav’ and have become figures of national misery or disgust. The term ‘chav’ has been described as the “ubiquitous term of abuse against the white poor” (Tyler, 2008, p. 17), which has been used to mock and deride the appearance, accent, clothing, lifestyle and culture of working class men and women in Britain (Tyler, 2008). The example of the “celebrity chav” indicates that the social class cannot be understood only from the point of view of economic capital and therefore offers a broad approach for consumer identity construction.

Many scholars suggest the presence of co-branding following from personal branding activities, for example between ordinary employees or CEOs and companies. Furthermore, due to spill-over and meaning transfer, deliberate and unintentional effects may arise between human brands, corporate brands, and product brands. For example, the entire artistic brand, from which consumers derive their judgements about the uncertain product quality of the artwork, results from spill-over effects between an artist's human brand and the artist's artwork (Moulard et al., 2014).

Some first indications that personal branding impacts society can be found, as e.g., David Bowie's societal and cultural relevance is also obvious “by sanctioning his homosexuality as an important socio-cultural statement and response to Britain's post-industrial decline” (Lindridge and Eagar, 2015, p. 23).

### Antecedents of Personal Branding

From today's point of view, the branding of individuals is an old practice that has produced numerous examples in human memory, such as Alexander the Great (Braudy, 1997) and savant Goethe (Bendisch et al., 2013). An analysis of 18th century auction records serves as an additional example and revealed that artists have always been branded as the prices for their artworks was determined by their reputation and status in society (Preece and Kerrigan, 2015). It is obvious that there has been a long history during which celebrity was attained through family relationships or achieved through talent (Rojek, 2012). However, the affair of Elizabeth Taylor and Richard Burton in 1963 has been identified as “an insightful turning point, marking a juncture whereby the public were seen to have become more interested in one particular celebrity's private life than her abilities as an actress” (Mills et al., 2015, p. 5). As such, contemporary personal branding has not just become more media-driven, complex, and multilayered. However, it finds its most effective antecedents only in the recent past. The joint impact of societal, economic, and technological developments provide three key areas that have given rise to the emergence of ubiquitous personal branding.

First, the development of the new world of work means a transformation from an industrial to an information-based economy, with the spread of neoliberal capitalism and increasing complexity. Massive changes and turbulences were caused by the mass layoffs of the 1970s, followed in the late 20th and early 21st century by “economic globalization, new arenas of competition, and rapidly evolving information technologies” (Lair et al., 2005, p. 311). As a result, powerful social norms and pressures that promised stability in uncertain environments have become unstable. Individuals could no longer depend on employers to be “guarantors of life-long employment and personal economic stability” (Philbrick and Cleveland, 2015, p. 182). Competition for jobs increased, as careers became unpredictable, not limited to a single job description, and as traditional job applications based on a curriculum vitae became insufficient. “Hiring, as a consequence of these changes, has become a matter of choosing potential employees who signal that they are managing themselves correctly, replete with expandable skills, useful alliances, and appropriate branding strategies” (Gershon, 2014, p. 288). People offering their abilities, skills, and performance are in competition with each other, not dissimilar to the competition between products or services for attention in saturated markets. This need for personal responsibility and individual differentiation seems accompanied or exemplified by the emergence of the figure of the entrepreneur. Hearn (2008, p. 201) states that “the overt practices of self-branding […] have their root in the rise of the networked organization and the entrepreneurial workplace” which is supported by other scholars (e.g., Gandini, 2016). Workers are encouraged to become enterprises in their own right in corporate employment or in a job application process. Thus, personal branding serves as a supportive tool in employment in times of neoliberal precariousness and as a “communicative response to economic uncertainty” (Lair et al., 2005, p. 309).

Second, various forms of media have developed alongside the rise of the idea of visibility as a key currency in life. The explosion of the Web 2.0 and social media offers continuously evolving platforms for an emerging attention economy that self-branding is directly related to. Multiple media outlets enable personal branding for everyone, e.g., by searching on Google, sharing via Facebook, networking on LinkedIn, broadcasting on YouTube, or linking via Twitter to access and contribute to the story of the individual self. A key academic contribution that is frequently cited is the investigation of self-presentation in personal web space by Schau and Gilly (2003). They see the link between sociologist Erving Goffman's “presentation of self in everyday life” (Goffman, 1956) with the computer-mediated environment in that “personal Web sites allow consumers to self-present 24/7 beyond a regional setting to the virtual world” (Schau and Gilly, 2003, p. 387), as building a digital self can be taken as par for the course. David Bowie became “the first artist in 1999 to release an album (‘The Hours’) through the Internet signifying Bowie's human brand innovation” (Lindridge and Eagar, 2015, p. 21). With the development from Web 1.0 to Web 2.0, online personal branding has mutated into an interactive and meaningful presence through the use of chat rooms, blogs, and other kinds of third-party sites. Ubiquity and user-friendliness, free and open access, crumbling technological barriers, and space for individuals are factors inviting self-expression and self-presentation—not least for purposes of personal branding. Broader audiences can be reached, irrespective of time or place, while branding in social media is migrating from being an exclusive business pursuit to allowing individuals to create their own unique virtual spaces. Consequently, cultural values change, with fame and attention gaining significant importance and people mutating into “gossip-hungry consumers” (Mills et al., 2015, p. 1). Thus, “personal branding reflects one logical reaction to the cultural and political economics of Web 2.0” (Gehl, 2011, p. 2).

Third, a new understanding of individualism developed as a countermovement to traditional collectivistic systems. Scholars claim that the symptoms and forms of individualism represent a reflection of the concept of humankind in its respective era. Under earlier forms of capitalism, for instance, workers provided their physical and mental capacities to the employer for a limited period of time each day. By contrast, in the era of neoliberalism, the individual now owns and treats herself/himself as a corporate business aiming to maintain her/his human capital, i.e., her/his collection of skills, assets, and alliances. The responsibility for self-fulfillment, self-reliance, self-sufficiency, self-actualization, and self-realization as fundamental psychological needs lies exclusively with the individual today, reinforced by the American myth. Realizing the American dream implies accepting a world of change and opportunities in which “you can create and recreate yourself so as to be the master of your own destiny” (Lair et al., 2005, p. 314). This understanding of individualism is located as a difference that began to exist historically only within a broader system from the 1860s to the 1880s, in the wake of the Civil War in the US. At that time, people began to understand themselves as individuals individualized by their place within the system (Michaels, 1989). To the same degree of development, trust is eroding in any all-embracing system of determined norms and values, as the quest for identity fails when applying traditional collectivistic interpretations. Therefore, practitioners postulate the process of self-managed self-improvement as the means of choice, and the self-help movement appears as a precursor of personal branding.

Additionally, scholars (e.g., Shepherd, 2005) have identified existential angst as a driver and a major selling proposition for personal branding by consultants and counselors. The individual has to cope with the inevitability of building her/his human brand, as the otherwise inescapable consequence is “being marginalized or left behind” (Harris and Rae, 2011, p. 14) and going “through a brand divorce” (Lair et al., 2005, p. 329). Fear of losing one's livelihood is attended by the fear of losing human brand ownership, since someone else will manage the human brand if the individual does not do so himself or herself.

Nonetheless, the antecedents of personal branding have to be determined in more detail for different cultures and societies, e.g., comparing the US and Europe or considering traditional collectivistic societies such as Japan.

**Figure 3:**
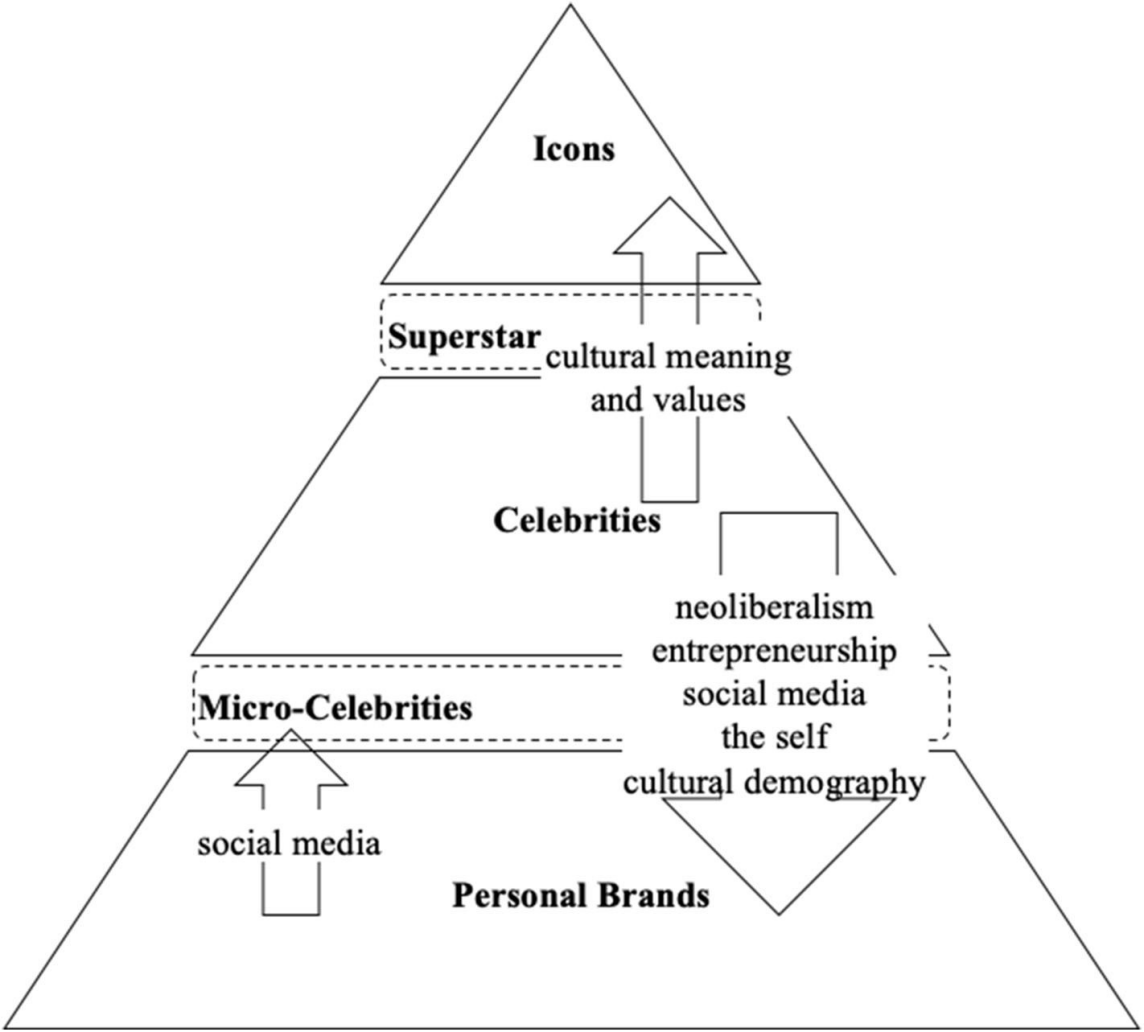
Pyramid of different human brand classes and their appearance in the history of personal branding.

Derived from the review of the classes of human brands and antecedents of the personal branding movement, celebrities appear as the cradle from which human brands for ordinary people have sprung, due to the opportunities and needs produced by societal, economic, and technological developments. Icons, in turn, represent a select group containing long-lasting and outstanding branded individuals who stand out from the growing number of commoditized celebrities (Figure 3).

### Key Ingredients and Applications

Beside numerous fragmented findings and concepts and some more general conceptual approaches, many scholars have come to agree on a small number of elements of what human brands may consist of and which fields of application they can be encountered in, partly based on empirical investigation.

#### Personality and Authenticity

At a very early stage of the personal branding process, one's personality, i.e., individual strengths and weaknesses, values, skills, expertise, and attributes, needs to be reflected in order to transform it into the human brand personality. Credibility and, in particular, authenticity are two attributes that are understood to represent the crucial ingredients for human brands. Authenticity affects attitudes toward the branded individual positively, which in turn is a critical component used in consumer judgments and decision-making. Consumers demonstrate an increased demand for authentic brands. An authentic human brand claims to represents the true self of the branded person and thus delivers a constant promise of value. Clarity as well as rarity, with its sub-dimensions of talent, discretion, and originality, contribute to authenticity and are closely linked to differentiation as a further key element of personal branding.

#### Differentiation

Most scholars tend to one-sidedly emphasize that the branded individual should differ from everybody else and, as such, neglect the points of parity (e.g., Chen, 2013). In contrast, Parmentier et al. (2013), who explored fashion models, identified points of differentiation to stand out from other competitors in terms of the amount and the quality of their field-specific cultural and social capital as well as points of parity that are visibly fitting in with the expectations of the field in which the human brand is competing. This is confirmed for first faculty positions in marketing as well (Close et al., 2011). Distinction does not necessarily have to have positive connotations. Even negative “bad boy” or “bad girl” images (Carlson and Donavan, 2013), the refusal to comply with societal conventions, or even scandals may lead to differentiation (Mills et al., 2015) and attention.

#### Visibility

Above all, visibility is named as indispensable, following the key expectation “You need to get noticed” (Gander, 2014, p. 101). On the one hand, a physical footprint is expected in the form of writing articles, speaking in public, extending one's network, giving presentations, or producing publications to create an offline self. On the other hand, a digital footprint leads to an online self by way of social media platforms, which obviously now plays a far more prominent role in personal branding. From another perspective, visibility is to be attained in two areas: First, on-field in the original field of practice and profession of the branded individual, aiming for instance for awards and honors conferred by peers in the field, and second, off-field outside of the professional field in the sense of building a mainstream media persona (Parmentier and Fischer, 2012). The interrelations of these different areas as well as constant connectivity with an increasing number of online opportunities suggests a need to bundle individual visibility activities in a transmedia model of storytelling and story-world construction. Stories that build brand meaning have transitioned from being developed by the original brand owner, i.e., storytelling, to being developed by the stakeholders, i.e., storygiving. The integration of different story elements that are dispersed across multiple media platforms in an episodic format and co-creative audience interaction are key characteristics (Elwell, 2014).

#### Narrative Identity

Contemporary ways of visibility and the digital world in particular call for a new paradigm to conceptualize the dialectic of the digital-analog self-identity. The self is much more actively managed, jointly constructed, interactive, openly disinhibited, confessional, multiply manifest, and influenced by what the branded individual and his or her avatars do online (Belk, 2013). Much of the influence on contemporary self-concepts and people's activities in creating them, is absent when only the self is studied offline in an extended way. It is not an either-or between offline and online identities, but an “as well” because “it becomes impossible to tell where one begins and the other ends as the two are seamlessly integrated. Online and off-line identities are not functionally equivalent to one another such that one is interchangeable for the other. Rather, together they co-create the experience of identity in the space between the digital and the analog” (Elwell, 2014, p. 235). The transmedia model (Elwell, 2014) serves as a helpful paradigm for understanding the nature of self-identity and self-formation in this new liminal space by offering the conceptual architecture necessary for exploring and articulating its integrated, dispersed, episodic, and interactive narrative character as a key element of the contemporary human brand. Integrated narrative elements, such literal series of episodes in the form of sequential films, books, and games create a continuous and evolving story of the self. In this respect, the psychological perspective contributes valuably to a deeper understanding of personal branding by defining the story a a selective reconstruction of the autobiographical past and a narrative anticipation of the imagined future that serves to explain, for the self and others, how the person came to be and where his or her life may be going (McAdams, 2011). Developing one's own human brand implies that the I becomes an author, seeking to fashion the Me into a self-defining story. Consequently, narrative identity is not just an internalized and evolving story of the self that provides a person's life with some semblance of unity, purpose, and meaning. Rather, narrative identity, then, is that feature of human selfhood that begins to emerge when the adolescent or young-adult I assumes the guise of a storyteller.

#### Social Media

Social media in particular are assumed to allow human brands to develop stronger bonds with consumers, resulting in “a formation of a social relations exchange” (Chen, 2013, p. 335) and a network of relationships in a general sense, as increased attention is a conditional element for brand attachment. In current digital knowledge industries, self-branding directly contributes to socialized value production through the social construction of a branded persona—a process called “digital work” (Gandini, 2016). In addition, specific consumer-brand relationships are built, for example in sports, where the athlete brand “has a positive influence on the extent to which consumers feel an emotional attachment to the athlete” (Carlson and Donavan, 2013, p. 204). Human brands to which people are attached offer potential as endorsers, which has been the primary focus when exploring athletes and celebrities as human brands. While autonomy, relatedness, and competence have been seen to serve as antecedents of the strength of people's attachment to human brands (Thomson, 2006), Loroz and Braig (2015) create an empirically more comprehensive and sophisticated picture of consumer attachments to human brands that emphasizes the dimension of competence.

#### Co-brands and Stakeholders

As human brands do not function in isolation, the collaborative process at work in building the human brand is obvious. Brand meaning transfer effects and co-creation are inevitable elements that are generally considered, be it between businesspeople or politicians and their organizations, athletes “fraternizing with figures from the entertainment world” (Parmentier and Fischer, 2012, p. 116), photographers and agencies and fashion models, or between employees and companies. Based on Freeman's (1984) stakeholder theory and the multi-stakeholder approach (Keller, 2003), stakeholder models of human brand equity are being developed for the art market, for CEO branding, and for professional rock climbers. First studies indicate beneficial as well as damaging co-branding activities in personal branding (e.g., Parmentier and Fischer, 2012) and a potential risk of broken links between human brands and organizational brands (e.g., Speed et al., 2015).

#### Brand Equity

After all efforts and investment into creating a human brand, it is crucial to measure whether personal branding activities are successful at all and, if so, to what extent they are. Certainly, the price of artworks, record sales or online downloads of a musician's work, career earnings, or the number of endorsement contracts as well as rankings in the annual Forbes's list of celebrities are measurable quantities, but they do not offer reliable information about the comparative human brand equity of different individuals. Professional equity that is built in the original field of practice and celebrity equity that is earned outside the original field are two main elements of human brand equity for football players (Parmentier and Fischer, 2012). A measurement scale for CEO human brands should contain work standards, style, leadership, personality, values, character, and teamwork (Chen and Chung, 2016) and CEO brand's characteristics and action parameters influence stakeholder's perceptions and behavior and may lead to the creation of added perceived value at reputational and financial level that reflects the actual essence of CEO brand equity (Cottan-Nir, 2019). Nonetheless, a real brand equity measurement in practice, considering all human brands as multidimensional constructs and taking into account the multi-stakeholder approach to co-create the human brands in a collective act, is still sorely lacking.

### Challenges for Personal Branding

By contrast to the rational or even enthusiastic contemplation of the consequences of personal branding, scholars (e.g., Lair et al., 2005; Gershon, 2014) are increasingly sensitive toward its dark side as well and have revealed its essential challenges.

“The more personal branding, the better the impact” does not necessarily work as expected either, as too successful a human brand may appear as a threat to colleagues or superiors in a corporate setting, resulting in suspicion and skepticism (Harris and Rae, 2011). Based on the optimal stimulation level (OSL) theory, consumers may switch quickly to other human brands due to their desire for variety. Too frequent encounters with a human brand may also cost stimulatory potential and may result in a perception of boredom (Huang et al., 2015). In same vein, a higher level of visibility increases the probability of getting involved in affairs compared to ordinary people and, especially with regard to online personal branding, professional and career advantages cannot be taken for granted. Inappropriate photos or information posted on a candidate's page, poor communication skills, “bad-mouthing” from former employers or fellow employees, implied links to criminal behavior, or confidential information about past employers are top areas of concern when seeking for job opportunities (Harris and Rae, 2011).

Scholars only sporadically point out the fundamental, gender-specific differences in the context of work and career in their investigations of personal branding. In this respect, the contemporary phenomenon of personal branding and all its advocates face the challenge of developing strategies to address two key issues. First, the common task in personal branding of combining one's own authenticity with the need to take on multiple roles shows significant differences between women and men in the way they cope with it. Women are expected to reach for the top, but also to look feminine, pay attention to their appearance, be there for their children and husbands and routinely take on the role of caretaker at work. Consequently, working women with families run the risk of experiencing even greater tension between work and family if they commit to becoming a human brand (Lair et al., 2005). In the sense of “true-to-self” strategies, women can maintain their authenticity as individuals yet still achieve the desired rewards if they are good enough (Fletcher, 1999; Singh et al., 2002). Really successful, however, are chameleons (usually males) who pick strategies out of a number of role models by trying different approaches, with a greater chance of understanding what worked for them (Singh et al., 2002). Additionally, it is not acceptable and is risky for future career progression to promote a “whole” identity in some organizations (Sheppard, 1989). Only a work-focused person receives the ticket for the next round. This limitation raises the question for women with children that energy must be spent on positioning themselves to fit into a model that they still consider “different.” Second, women are less likely to self-promote than men (Dobbins et al., 1990; Oakley, 2000; Singh et al., 2002). This gender gap in self-promotion is reflective of the gender gap in self-evaluations and, in addition, the gender gap in self-evaluations is specific to evaluations of own performance (Exley and Kessler, 2019). Women evaluate their performance less favorably than men, which then is likely to have a continuing impact on their careers. In contrast, men are actively reading the promotion systems in their organization and working to fit the career success model using impression management. Most of the managerial and professional males seem to understand and comply with the rules of the game of acknowledgment, recognition and promotion in a more straightforward and less emotional way compared to their female colleagues. Although many women are aware of the potential of impression management, self-expression and networking, they decide not to use it (Singh et al., 2002).

Authenticity represents a crucial ingredient for human brands as it affects attitudes toward branded individuals positively, which in turn is a critical component used in consumer judgments and decision-making (Mills et al., 2015). The understanding of what authenticity of a human brand means exactly is predominantly 2-fold. On the one hand, it is understood as the “fit between persona and underlying personality” (Speed et al., 2015) and to act “according to his/her true self” (Moulard et al., 2015). In this way, authenticity is thought to be derived from intrinsic motivation as opposed to extrinsic motivation, with commercially driven interests which implies that commercialization must not be part of intrinsic motivation. Against this background, Paris Hilton, for example, is perceived as hardly authentic, but she is without doubt a celebrity brand (Moulard et al., 2015). Is authenticity then indeed indispensable in personal branding? On the other hand, the focus lies on being “unconventional and […] seen to be going against the mainstream” (Lunardo et al., 2015). Here, clarity as well as rarity contribute to authenticity and are closely linked to differentiation as a further key element of personal branding. However, distinction does not necessarily have to have positive connotations. Even negative “bad boy” or “bad girl” images (Carlson and Donavan, 2013), the refusal to comply with society's conventions, or even scandals may lead to differentiation. How to separate then between “good authenticity” and “bad authenticity,” and how far does a human brand benefit from it?

Obviously, a dilemma for personal branding arises from its tendency to demand both maintaining the true self, i.e., authenticity, and responding to different target groups, even more when it comes to creating a digital footprint that implies multiple online identities. In branding the self, people often have trouble crafting their individual web presence across various platforms when fashioning a coherent branded self (Gershon, 2014). Social psychologist Gergen (1991) and other postmodernists have argued that multiple selves are an adaptive response to a world of multiple demands. The multiplicity of roles is ascribed to represent a major psychological challenge today as people are expected to enact different identities to fit in different contexts (Leary and Tangney, 2012), which is in line with scholars' perspective on personal branding, especially considering human brands' presence on social media: “They struggle to seem like a coherent self across multiple platforms, despite the complexities of audiences for the different interfaces they use” (Gershon, 2014, p. 29). In fact, scholars indicate successful examples of human brands consisting of different roles offline as well as online, such as David Beckham and David Bowie. Beckham's human brand, for example, encompasses several masculinities, including the romantic and compassionate husband, the hands-on father, the football legend and the fashion icon. He has become what his fans wish to see in him, which suggests that an important component of his popularity and success derives from these multiple identities (Cashmore and Parker, 2003; Vincent et al., 2009; Cocker et al., 2015). The human brand of late celebrity David Bowie consisted of three components, i.e., the real person (David Jones), the performance persona (David Bowie) and the characters derived from this persona, such as Ziggy Stardust (Lindridge and Eagar, 2015). Despite a few successful examples in the celebrity sector, there remains the question of the effects when having multiple discrepant identities for the infinite number of human brands. Psychologists found that, despite their buffering effects in stressful events (Linville, 1987), a greater variability across identities was associated with lower well-being (Donahue et al., 1993), a lack of coherence and integrity (Ryan et al., 2005), and inauthenticity (Sheldon et al., 1997). Self-determination theory could serve as a helpful framework as “under conditions in which the identities offered individuals are both supported by significant others and allow fulfillment of the psychological needs for relatedness, competence, and autonomy, a healthy integration of the individual is possible” (Leary and Tangney, 2012, p. 242).

Despite the creation of few personal branding frameworks, partly based on empirical studies (e.g., Preece and Kerrigan, 2015) and partly as a result of conceptual work (e.g., Bendisch et al., 2013), a comprehensive personal branding framework or even theory has not yet been developed in the academic field. Even in a well-defined field such as commercial sports, a general model for personal branding is not effective, as “wrestlers or boxers might be seen as rude athletes, while golfers might be seen as sophisticated ones” (Lunardo et al., 2015, p. 706). Nonetheless, the empirically based artistic brand model constructed as a diffusion process over time (Preece and Kerrigan, 2015) may serve as an inspiring example. Broken down to the individual level, an infinite number of different human brands is possible, with each having its own human complexity. In addition, the more people have acquired a status symbol as a human brand, the less distinctive it is and the less status it confers on its holders. Simply said, “even if it were possible that we could all be famous, if everyone were famous, then no one would be famous” (Holmes and Redmond, 2006, p. 14).

As personal branding represents the logical extension of previous forms of branding, such as product brands, service brands, corporate brands, or retail brands, it would seem natural to call for an application of traditional branding practices in equal measure to the younger field of personal branding. As a matter of fact, this transfer has few clear advocates (e.g., Close et al., 2011; Ternès et al., 2014) or critics (e.g., Russell and Schau, 2010; Preece and Kerrigan, 2015), but it promises constructive approaches (e.g., Parmentier and Fischer, 2012; Preece and Kerrigan, 2015) that very selectively adapt proven branding practices. So far, no one attribute from traditional branding can be identified that has explicitly been rejected from personal branding. Others, such as the brand personality (Aaker, 1997), competition which implies points of differentiation as well as points of parity (Keller et al., 2002), brand visibility (Keller, 2013), brand relationships (Fournier, 1998), or brand meaning transfer (McCracken, 1989) as well as brand co-creation (Prahalad and Ramaswamy, 2000) have already been adapted to the context of personal branding as discussed earlier, defining them as single key ingredients. Nonetheless, personal branding is not investigated as an entire process to clarify how it emerges and even though a “great brand is not built by accident” (Keller, 2013, p. 125), it remains unclear how aware people really are about their own human brand and their brand building process. Furthermore, target groups and categories represent two indispensable dimensions in traditional branding (Keller, 2013). However, abstract terms such as “customer” (Gehl, 2011), “consumer” (Carlson and Donavan, 2013), and “audience” (Mills et al., 2015) are widely applied in personal branding but it remains nebulous as to who is meant by this. Similarly, the term “target market” serves as an undefined focus for numerous activities in human brand positioning (Shepherd, 2005), except for the art market (Schroeder, 2005), the music market (Lindridge and Eagar, 2015) and the job market (Zamudio et al., 2013). Finally, the dimension of time suggests that “if there is one rule for modern branding, however, it is that brands can never stand still” (Keller, 2013, p. 479). One should understand that “achieving and maintaining your personal brand is a journey, not a destination” (Trepanier and Gooch, 2014, p. 57). Human brands are not static and face continuous change during their lifetimes. Athletes may experience unexpected injuries or performance slumps (Arai et al., 2014), models have biological limits affecting their ability to keep their physical appearance (Parmentier et al., 2013), transgressions can damage human brands (Moulard et al., 2015) and, finally, every branded individual will pass away (Fillis, 2015). There are first insights into viable means to extend the life expectancy of a human brand, as can be seen in David Beckham maintaining human brand equity even after his active career in football had ended (Parmentier and Fischer, 2012). Similarly, the artistic brand model has been considered in terms of a diffusion process over time (Preece and Kerrigan, 2015). However, the research domains of traditional branding (Kapferer, 2012; Keller, 2013) as well as personal branding (Philbrick and Cleveland, 2015) show clear agreement about brands having to be managed over time. But, in contrast to traditional branding again, the issue of longevity faces a lack of clarity too in the sense of how to handle it in personal branding.

## Conclusions

Fundamentally, personal branding has long outgrown its original academic role as another instance of “broadening the concept of marketing” (Kotler and Levy, 1969). Instead, it is worth appreciating personal branding as a distinct and interdisciplinary expression of branding and not just as a simple variation thereof. However, before giving a positive answer to the key question of whether science can “reclaim self-marketing and personal branding from the enthusiasts” (Shepherd, 2005, p. 12), further academic efforts are needed. Beside empirical studies, different formats such as review papers (Gorbatov et al., 2018) not only offer valuable contributions in this regard, but they also serve as a means to incite the required sophisticated debate on the contemporary phenomenon of human brands and their emergence.

Universally valid personal branding frameworks or even theories cannot be identified yet, and those that have been put forward do not show great promise due to their fragmented nature. Therefore, this review suggests updated definitions to better structure the fragmented approaches toward the process of personal branding and to the human brand as a thing, as proposed above. Celebrities serve as the cradle of the personal branding movement as well as for all kind of human brands (Figure 3). Apart from the “celebrity,” two additional classes of human brands, i.e., the “icon” and the “personal brand,” as well as two intermediate classes, i.e., the “superstar” between celebrities and icons and the “micro-celebrity” between celebrities and personal brands, complement the aspect of classes.

Nonetheless, personal branding happens in many and diverse shapes and forms and takes place in a distinctly complex setting, so a precise and readily transferable recipe for personal branding that is applicable to every walk of life has to remain wishful thinking. Any search for the one universal personal branding theory would seem doomed from the outset. In addition, since proposing a model implies pragmatism, structure, and universality, personal branding faces a paradox in that a generalizable branding model has to be applied to something that is completely unique, namely human beings. Therefore, more empirical evidence, exploration, and conceptual development are sorely needed, as they may result in class and category-specific definitions as well as models. In particular, the icon as a human brand class, celebrity academics, or the aristocracy call for deeper investigation, while social media influencers, e.g., Bhad Bhabie, as the new type of endorsers and “celefictions” (Nayar, 2009; Kerrigan et al., 2011), such as Harry Potter, Lara Croft or Dr Z, must not be neglected.

Gender differences in personal branding, whether in the branded individual itself or on the consumer side, have so far only been examined sporadically and rather one-sidedly with regard to the effect of human brands' gendering in social media (e.g., Duffy and Pruchniewska, 2017; Draper and McDonnell, 2018). But the open questions are far more fundamental and very diverse, for example with regard to the world of work. What explains the existence of the gender gap in self-evaluations which affects the gender gap in self-promotion? How can the gender gap in self-evaluations be mitigated? how does the potential for gender-specific backlash influence self-evaluations and how employers view self-evaluations? (Exley and Kessler, 2019). Future research should also consider the gender-oriented role of personal branding in private life. Sexual selection theory, for instance, can help to understand how people act in an effort to attract another person (Schmitt and Buss, 1996) and psychological mechanisms, to suggest further possibilities, appear to underlie between-sex differences in what people prefer in mates (Buss, 1989) and how they attract mates (Buss, 1988).

Especially since, looking beyond the snapshot, the sustainability of human brands still suffers from a lack of attention, research questions such as “How do top managers' human brands emerge over time?” demand an answer based on empirical studies. From an academic vantage point, this more comprehensive understanding of personal branding also needs to expand from the synchronic to the diachronic level, that is, the human brand's fate over time in the sense of developing a lifecycle approach and identifying ways to ensure the longevity of a brand. The same applies as well to further branding attributes promising useful applicability to personal branding such as target groups, competition, visibility, or human brand authenticity or, finally, human brand equity measurement.

As human brands cannot function in isolation, brand leveraging processes between human brands and their organizational environment and stakeholders need to be investigated further. How do human brands develop an interactive, individualized, yet communal brand experience at all brand touch points for all stakeholders, considering that not all stakeholders are actively involved?

All in all, it is obvious that personal branding is an interdisciplinary domain where research into branding-oriented explanatory and development approaches is given considerable, if not too much, emphasis. Although some scholars already refer to psychological models and theories in their research, we advocate for much more attention to be paid to the components “personal” and “human” in personal branding and human brand. The concept of narrative identity, for instance, plays a major role today in the multi-layer personality theory developed by McAdams and Pals (2006) which corresponds to today's “flexible personality” the modalities of selfhood have shifted to from a preoccupation with “character” in the19th century to “personality” in the 20th (Hearn, 2008). Additionally, the concept of narrative identity serves as a framework to understand how human beings make narrative sense out of their own lives, how they develop the stories that come to comprise their very identities, how those stories change over time, and how those stories function—psychologically, socially, morally, culturally—as the storyteller journeys across the long course of adult life (McAdams, 2011). In turn, personal brands have so far been presented primarily as a static construct, which must be overcome in the future through a life-span approach. The method of process research, which has proven itself in organizational research, is just as obvious in its application as models and concepts from psychology. Erikson's (1980) model of identity development, for instance, provides different life stages each with its own central identity tasks that can contribute to the emergence of a human brand over time. Especially for the further challenges that personal branding faces, applied psychology offers numerous options for a deeper exploration of this contemporary phenomenon. A review from a psychological perspective, for instance, examining the literature on the context in which the concept of self-branding developed, the experience of presenting self-brands to a public audience, and the psychological construction of authenticity within the self-branding discourse, would certainly contribute significantly to the state of knowledge on personal branding.

This review provides an overview of the contemporary phenomenon of personal branding from the angle of academic publications. As it certainly cannot avoid certain shortcomings, a deeper and even more systematic literature research is recommended, which, for example, implies specific inclusion as well as exclusion criteria (Ramírez et al., 2017), such as the classification of journals or a more recent timeslot for the articles' publication.

In the end, personal branding remains a field deserving to be scholarly explored and an academic impulse for rethinking branding, as it may sensitize scholars in applied psychology to the concept of more collaboration with practitioners and with other academic domains, e.g., culture theory, management education, organizational studies, or vocational behavior, in the interest of knowledge dissemination and mutual enrichment.

## Data Availability Statement

The original contributions presented in the study are included in the article/supplementary material, further inquiries can be directed to the corresponding author/s.

## Author Contributions

SS is the main author of the submitted paper. JH initiated this manuscript and co-developed key visuals. CG contributed to the methodology and the positioning. All authors contributed to the article and approved the submitted version.

## Conflict of Interest

The authors declare that the research was conducted in the absence of any commercial or financial relationships that could be construed as a potential conflict of interest.
