# Supplementary material for: Old Practice, but Young Research Field: A Systematic Bibliographic Review of Personal Branding
Source: Front Psychol. 2020 Aug 11;11:1809. doi: 10.3389/fpsyg.2020.01809 (PMC7433337; doi:10.3389/fpsyg.2020.01809)
Supplement: Supplementary file 1 [file Data_Sheet_1.docx]

**Appendix 1:** Overview of publications concerning three different classes of personal brand in eleven different categories

| **Categories** | **Sub-categories** | **Classes of Human Brands** | | |
| --- | --- | --- | --- | --- |
|  |  | **Icon** | **Celebrity** | **Personal brand** |
| **Sports** | Athletes | --- | Rascher et al., 2017; Sassenberg et al., 2012; Shuart, 2007; Summers & Morgan, 2008 | Agyemang, 2011; Arai et al., 2013; Arai et al., 2014; Ballouli & Hutchinson, 2012; Carlson & Donavan, 2013; Carlson & Donavan, 2017; Chang, 2018; Chang et al., 2018; Constantinescu, 2017; Desmarais, 2017; Emmons & Mocarski, 2014; Geurin, 2017; Geurin-Eagleman & Burch, 2016; Green, 2016; Hasaan et al., 2016; Hasaan et al., 2018; Hasaan et al., 2019; Hodge & Walker, 2015; Kunkel et al., 2019; Lebel & Danylchuk, 2014; Lobpries et al., 2017; Lobpries et al., 2018; McGhee, 2012; Parris et al., 2014; Schwartz & Vogan, 2017; Staskeviciute-Butiene, 2014; Tsiotsou, 2016; Williams et al., 2015; Wilson & Liu, 2012; Zhou & Tainsky, 2017 |
|  | Athletic trainers | --- | --- | Kahanov & Andrews, 2001; Walsh & Williams, 2017 |
|  | Athletes from specific disciplines | David Beckham (Kelting & Rice, 2013; Parmentier & Fischer, 2012) | Ryan Giggs (Parmentier & Fischer, 2012)  LeBron James (Fresco, 2017)  Andy Murray (Davies & Slater, 2015)  Suzann Pettersen (Kristiansen & Williams, 2015)  Eugen Sandow (Morais, 2013)  Annika Sörenstam (Cortsen, 2013)  Tiger Woods (Tanner & Maeng, 2012) | Bigsby et al., 2019; Chadwick & Burton, 2008; Kakitek, 2018; Lebel & Danylchuk, 2012; O'Reilly & Braedley, 2008; Price et al., 2013 |
| **Academics** | General | --- | --- | Banet-Weiser & Juhasz, 2011; Borman-Shoap et al., 2019; Close et al., 2011; Cole-Turner, 2019; Duffy & Pooley, 2017; Hotez, 2018; Radford et al., 2018; Reif-Lehrer, 1992; Shafaei et al., 2019 |
|  | Professors | --- | --- | Jillapalli & Jillapalli, 2014; Jillapalli & Wilcox, 2010; Zamudio et al., 2013; Zamudio & Meng, 2015 |
|  | Students | --- | --- | Bergh et al., 2017; Bronstein, 2014; Chakrabarti, 2014; Edmiston, 2014; Flostrand et al., 2016; Holmberg & Strannegård, 2015; Hood et al., 2014; Humphrey et al., 2019; Ilies, 2018; Johnson, 2017; Jones & Leverenz, 2017; Lee & Cavanaugh, 2016; Levin et al., 2019; Manai & Holmlund, 2015; McCorkle et al., 1992; McCorkle et al., 2003; Myers, 2017; Robson, 2019; Shuker, 2014; Stanton & Stanton, 2013; Taylor, 2003; Watson, 2019 |
| **Politicians** | General | --- | McKernan, 2011; Street, 2004 | Algara, 2019; Bors, 2019; Coesemans & De Cock, 2017; Colliander et al., 2017; Jones, 2010; Medveschi & Frunzã, 2018; Milewicz & Milewicz, 2014; Speed et al., 2015 |
|  | Prime ministers | --- | Tony Blair (Scammell, 2007)  Benito Mussolini (Swan, 2016)  Kevin Rudd (Burgess et al., 2017; Craig, 2014) | --- |
|  | Presidents | --- | George W. Bush (Tanner & Maeng, 2012)  Wladimir Putin (Campbell & Denezhkina, 2017)  Donald Trump (Hearn, 2016; Pérez-Curiel & Naharro, 2019) | --- |
|  | Election candidates | --- | Charles Kennedy (Harris & Lock, 2001) | Chai & Kim, 2013; Kaneva & Klemmer, 2016; Parker, 2012; Van Steenburg & Guzmán, 2019 |
|  | Ordinary politicians | --- | --- | Rozanova, 2017 |
| **Visual artists** | General | --- | --- | Baumgarth & O’Reilly, 2014; Baumgarth et al., 2014; Hernando & Campo, 2017; Kucharska & Mikolajczak, 2018; Moulard et al., 2014; Preece & Kerrigan, 2015; Schroeder, 2005 |
|  | Painters/Graphic artists | --- | Thomas Kinkade (Fillis, 2015)  Ernst Ludwig Kirchner (Weikop, 2012)  Pablo Picasso (Muñiz Jr. et al., 2014)  Andy Warhol (Kerrigan et al., 2011) | --- |
|  | Sculptors | --- | Ai Weiwei (Preece, 2015) | --- |
|  | Video/film producers | --- | Marina Abramovic (Marcus, 2015) | --- |
| **Performing artists** | Actors | --- | Drew Barrymore (Choi & Rifon, 2012)  Russell Brand (Mills et al., 2015)  Dustin Hoffmann (Tripp et al., 1994)  Elizabeth Hurley (Barron, 2007)  Jena Malone (Wohlfeil & Whelan, 2012)  Julia Roberts (Choi & Riffon, 2012) | Mathys et al., 2016 |
|  | Musicians | David Bowie (Lindridge & Eagar, 2015; Eagar & Lindridge, 2015) | Cheryl Cole (Cocker et al., 2015)  Ladi6 (Friend, 2015)  Lady Gaga (Click et al., 2013)  Kylie Minogue (Chapman et al., 2005) | Huang & Huang, 2016; Matenge, 2013; Meiseberg, 2014; Turri et al., 2013; Saboo et al., 2016; Scharff, 2015 |
|  | Comedians | --- | Matthew Broderick (Tripp et al., 1994) | --- |
|  | Models | --- | Tyra Banks (Keller, 2014; Persis Murray, 2015)  Katie Price (Cocker et al., 2015; Genz, 2015) | Belk, 2019; Parmentier et al., 2013 |
|  | TV Anchor | --- | Bethenny Frankel (Nayar, 2015)  Oprah Winfrey (Loroz & Braig, 2015) | Finneman et al., 2019 |
| **Aristocracy** | Royals | British royals (Otnes & MacIaran, 2018) | --- | Dionise, 2018 |
| **Producers of hedonic products** | Chefs | --- | Chen et al., 2017; Clark et al., 2016; Ehrmann et al., 2009 | --- |
| **Professional services** | General | --- | --- | Abrate & Viglia, 2019; Henning & Padayachee, 2018; Maiksteniene, 2009; Ogutu & Ougo, 2016; Shek et al., 2015; Tussvadiah & Park, 2018 |
|  | Medical staff | --- | --- | Brigham, 2016; Cederberg, 2017; Chu et al., 2018; Ioan et al., 2014; Kalia et al., 2017; Luca et al., 2015; Marwitz et al., 2018; Mishra, 2019; Munden, 2015; Trepanier & Gooch, 2014 |
|  | Consultants | --- | --- | Pagis & Ailon, 2017 |
|  | IT professionals | --- | --- | Johnson, 2015 |
|  | Engineers | --- | --- | Brennan et al., 2015; Sheikh & Lim, 2011 |
|  | Salespeople | --- | --- | Amoako & Okpattah, 2018; Little, 2012 |
|  | Teachers | --- | --- | Ilina et al., 2017 |
|  | Librarians | --- | --- | Ahmad et al., 2016; Eke, 2012; Gall, 2010; Kalbande, 2019; Schneider, 2012; Thomas, 2011 |
| **Self-employed** | General | --- | --- | Gandini, 2016 |
|  | Creative industry | --- | --- | Duffy & Pruchniewska, 2017; Pick et al., 2015; Pruchniewska, 2018 |
|  | Spiritual | --- | --- | Gregory, 2019 |
| **Business managers** | CEOs | --- | --- | Alghawi et al., 2014; Bendisch et al., 2013; Catellani et al., 2016; Chen & Chung, 2016; Chen & Chung, 2017; Cottan-Nir, 2019; Cottan-Nir & Lehman-Wilzig, 2018; Erdoğmuş & Esen, 2018; Fetscherin, 2015; Friel & Duboff, 2009; Takács et al., 2018; Mudambi et al., 2019; Scheidt et al., 2018; Treadway et al., 2009; Wade et al., 2006 |
|  | Executives | --- | --- | Karaduman, 2013; Schlosser et al., 2017 |
|  | Leaders | --- | --- | Manurung, 2015; Nolan, 2015; Uymaz, 2016 |
|  | Entrepreneurs | --- | Martha Stewart (Fournier, 2010; Murphy, 2010) | Ekhlasi et al., 2015; Raftari & Amiri, 2014; Razeghi et al., 2016; Sweeney et al., 2018; Thompson-Whiteside et al., 2018 |
|  | Owner-manager | --- | --- | Resnick et al., 2016 |
| **Content creators** | Journalists | --- | --- | Bossio & Sacco, 2017; Brems et al., 2017; Bruns, 2012; Canter, 2015; Carpenter et al., 2017; Hanusch, 2018; Hanusch & Bruns, 2017; Hedman, 2015; Hedman & Djerf-Pierre, 2013; Holton & Molyneux, 2017; Jukes, 2019; Molyneux, 2015; Molyneux, 2019; Molyneux & Holton, 2015; Molyneux et al., 2018; Molyneux et al., 2019; Olausson, 2017; Olausson, 2018; Ottovordemgentschenfelde, 2017; Schultz & Sheffer, 2012; Van Hove et al., 2018; Zeng & Song, 2018 |
|  | Authors | --- | Elizabeth Gilbert (Johns & English, 2016) | Bremner & Phung, 2015; Corrigan, 2015; Makkai, 2016 |
|  | Influencers | --- | Kim Kardashian (Harvey, 2018) | Khamis et al., 2017 |
|  | Bloggers | --- | --- | Ahmad et al., 2013; Archer, 2019; Bronstein, 2013; Delisle & Parmentier, 2016; Draper & McDonnell, 2018; Duffy, 2015; Duffy & Hund, 2015; Erz & Christensen, 2018; Hendrawan &Nahdiah, 2019; Kretz & De Valck, 2010; Liu & Suh, 2017; McFadden, 2018; Pihl, 2013; Safitri, 2017; Van Nuenen, 2016; Wang et al., 2015 |
|  | YouTubers | --- | Jenna Marbles (Maguire, 2015) | Chen, 2013; Harrington, 2019; Lovelock, 2017; Smith, 2014; Tarnovskaya, 2017 |
|  | Vloggers | --- | Bubz (García-Rapp & Roca-Cuberes, 2017)  Zoe Sugg (Berryman & Kavka, 2017) | Berryman & Kavka, 2017; Pahwa, 2019 |

**References**

Abrate, G., and Viglia, G. (2019). Personal or product reputation? Optimizing revenues in the sharing economy. *Journal of Travel Research*. 58, 136-148. doi.org/10.1177/0047287517741998

Agyemang, K. J. (2011). Athlete brand revitalisation after a transgression. *Journal of Sponsorship.* 4.

Ahmad, R., Hashim, L., and Harun, N. (2016). Criteria for effective authentic personal branding for academic librarians in Universiti Sains Malaysia libraries. *Procedia-Social and Behavioral Sciences*. 224, 452-458. doi.org/10.1016/j.sbspro.2016.05.420

Ahmad, A. L., Bromley, M., and Cokley, J. (2013). The social reality of blogging and empowerment among Malaysian bloggers. *Journal of Asian Pacific Communication*. 23, 210-221. doi.org/10.1075/japc.23.2.03ahm

Algara, C. (2019). The conditioning role of polarization in US senate election outcomes: A direct-election era & voter-level analysis. *Electoral Studies*. 59, 1-16. doi.org/10.1016/j.electstud.2019.02.006

Alghawi, I. A., Yan, J., and Wei, C. (2014). Professional or interactive: CEOs’ image strategies in the microblogging context. *Computers in Human Behavior*. 41, 184-189. doi.org/10.1016/j.chb.2014.09.027

Amoako, G. K., and Okpattah, B. K. (2018). Unleashing salesforce performance: The impacts of personal branding and technology in an emerging market. *Technology in society*. 54, 20-26. doi.org/10.1016/j.techsoc.2018.01.013

Arai, A., Ko, Y. J., and Kaplanidou, K. (2013). Athlete brand image: scale development and model test. *European Sport Management Quarterly*. 13, 383-403. doi.org/10.1080/16184742.2013.811609

Arai, A., Ko, Y. J., and Ross, S. (2014). Branding athletes: exploration and conceptualization of athlete brand image. *Sport Management Review.* 17, 97-106. doi.org/10.1016/j.smr.2013.04.003

Archer, C. (2019). How influencer ‘mumpreneur’ bloggers and ‘everyday’ mums frame presenting their children online. *Media International Australia*. 170, 47-56. doi.org/10.1177/1329878x19828365

Ballouli, K., and Hutchinson, M. (2012). Branding the elite professional athlete through use of new media and technology: an interview with Ash De Walt. *International Journal of Entrepreneurial Venturing*. 4, 58-64. doi.org/10.1504/ijev.2012.044818

Banet-Weiser, S., and Juhasz, A. (2011). Feminist labor in media studies/communication: Is self-branding feminist practice?. *International Journal of Communication*. 5, 1768-1775.

Barron, L. (2007). The habitus of Elizabeth Hurley: Celebrity, fashion, and identity branding. *Fashion Theory*. 11, 443-461. doi.org/10.2752/175174107x250244

Baumgarth, C., and O’Reilly, D. (2014). Brands in the arts and culture sector. *Arts Marketing: An International Journal*. 4, 2-9. doi.org/10.1108/am-08-2014-0028

Baumgarth, C., O'Reilly, D., Sjöholm, J., and Pasquinelli, C. (2014). Artist brand building: towards a spatial perspective. *Arts Marketing: An International Journal*. 4, 10-24. doi.org/10.1108/am-10-2013-0018

Belk, R. (2019). On standing out and fitting in. *Journal of Global Fashion Marketing*. 10, 219-227. doi.org/10.1080/20932685.2019.1615527

Bendisch, F., Larsen, G., and Trueman, M. (2013). Fame and fortune: a conceptual model of CEO brands. *European Journal of Marketing.* 47, 596-614. doi:10.1108/03090561311297472

Bergh, L., Jordaan, J., Lombard, E., Naude, L., and van Zyl, J. (2017). Social Media, Permanence, and Tattooed Students: The Case for Personal, Personal Branding. *Critical Arts*. 31, 1-17. doi.org/10.1080/02560046.2017.1345972

Berryman, R., and Kavka, M. (2017). ‘I Guess A Lot of People See Me as a Big Sister or a Friend’: the role of intimacy in the celebrification of beauty vloggers. *Journal of Gender Studies*. 26, 307-320. doi.org/10.1080/09589236.2017.1288611

Bigsby, K. G., Ohlmann, J. W., and Zhao, K. (2019). Keeping it 100: Social Media and Self-Presentation in College Football Recruiting. *Big data*. 7, 3-20. doi.org/10.1089/big.2018.0094

Borman-Shoap, E., Li, S. T. T., St Clair, N. E., Rosenbluth, G., Pitt, S., and Pitt, M. B. (2019). Knowing Your Personal Brand: What Academics Can Learn From Marketing 101. *Academic Medicine*. 94, 1293-1298. doi.org/10.1097/acm.0000000000002737

Bors, O. C. (2019). The Importance of Image when Developing a Powerful Political Brand. *Postmodern Openings*. 10, 72-85. doi.org/10.18662/po/82

Bossio, D., and Sacco, V. (2017). From “selfies” to breaking Tweets: How journalists negotiate personal and professional identity on social media. *Journalism practice*. 11, 527-543. doi.org/10.1080/17512786.2016.1175314

Bremner, S., and Phung, B. (2015). Learning From the Experts: An Analysis of Résumé Writers' Self-Presentation on LinkedIn. *IEEE Transactions on Professional Communication*. 58, 367-380. doi.org/10.1109/tpc.2016.2519319

Brems, C., Temmerman, M., Graham, T., and Broersma, M. (2017). Personal Branding on Twitter: How employed and freelance journalists stage themselves on social media. *Digital Journalism*. 5, 443-459. doi.org/10.1080/21670811.2016.1176534

Brennan, R., Halliday, S., Sheikh, A., and Lim, M. (2015). The making of brand attachment and brand meanings: the case of a UK engineering services firm. *Marketing Intelligence & Planning*. 33, 887-907. doi.org/10.1108/mip-06-2014-0106

Brigham, T. J. (2016). Online professional profiles: Health care and library researchers show off their work. *Medical reference services quarterly*. 35, 440-448. doi.org/10.1080/02763869.2016.1220760

Bronstein, J. (2014). Creating possible selves: information disclosure behaviour on social networks. *Information Research: An International Electronic Journal*. 19, n1.

Bronstein, J. (2012). Being private in public: information disclosure behaviour of Israeli bloggers. *Information Research: An International Electronic Journal*. 18, n1.

Bruns, A. (2012). Journalists and Twitter: How Australian news organisations adapt to a new medium. *Media International Australia.* 144, 97-107. doi:10.1177/1329878X1214400114

Burgess, J., Spinks, W., and Sharma, B. (2017). The Effect of a Brand Transgression on a Politician’s Brand Resonance: The Case of Kevin Rudd. *Journal of Political Marketing*. 1-24. doi.org/10.1080/15377857.2017.1407386

Campbell, A., and Denezhkina, E. (2017). From project Putin to brand Putin. *Celebrity Studies*. 8, 318-323. doi.org/10.1080/19392397.2017.1311511

Canter, L. (2015). Personalised tweeting: The emerging practices of journalists on Twitter. *Digital Journalism*. 3(6), 888-907. doi.org/10.1080/21670811.2014.973148

Carlson, B. D., and Donavan, D. T. (2013). Human brands in sport: athlete brand personality and identification. *Journal of Sport Management.* 27, 193-206. doi.org/10.1123/jsm.27.3.193

Carlson, B. D., and Donavan, D. T. (2017). Be Like Mike: The Role of Social Identification in Athlete Endorsements. *Sport Marketing Quarterly*. *26*, 176-191.

Carpenter, S., Kanver, D., and Timmons, R. (2017). It’s about me: A study of journalists’ self-presentation of their visual and verbal selves. *Journalism Practice*. 11, 1246-1266. doi.org/10.1080/17512786.2016.1245587

Catellani, A., Zerfass, A., Verčič, D., and Wiesenberg, M. (2016). Managing CEO communication and positioning. *Journal of Communication Management*. 20, 37-55. doi.org/10.1108/jcom-11-2014-0066

Cederberg, C. D., (2017). Personal branding for psychologists: Ethically navigating an emerging vocational trend. *Professional Psychology: Research and Practice*. 48, 183-190. doi.org/10.1037/pro0000129

Chadwick, S., and Burton, N. (2008). From Beckham to Ronaldo--Assessing the nature of football player brands. *Journal of Sponsorship*. 1, 307-317.

Chai, Y. D., and Kim, J. S. (2013). Mediator Effect of Presidential Candidate Brand Affiliated to Certain Party. *Journal of the Korea Safety Management and Science*. 15, 303-315. doi.org/10.12812/ksms.2013.15.1.303

Chakrabarti, D. (2014). Awareness and importance of ‘personal branding’phenomenon among post-graduate management students: An empirical enquiry. *International Journal of Research and Development in Technology and Management Science*. 21, 88-101.

Chang, Y. (2018). When infamy becomes fame: The positive side of negative athlete publicity. *Journal of Sport Management*. 32, 401-411. doi.org/10.1123/jsm.2017-0082

Chang, Y., Ko, Y. J., and Carlson, B. D. (2018). Implicit and explicit affective evaluations of athlete brands: The associative evaluation-emotional appraisal-intention model of athlete endorsements. *Journal of Sport Management*. 32, 497-510. doi.org/10.1123/jsm.2017-0271

Chapman, S., Holding, S., McLeod, K., and Wakefield, M. (2005). Impact of news of celebrity illness on breast cancer screening: Kylie Minogue's breast cancer diagnosis. *Medical Journal of Australia*. 183, 247-250. doi.org/10.5694/j.1326-5377.2005.tb07029.x

Chen, C.-P. (2013). Exploring personal branding on YouTube. *Journal of Internet Commerce.* 12, 332-347. doi:10.1080/15332861.2013.859041

Chen, H. M., and Chung, H. M. (2016). How to Measure Personal Brand of a Business CEO. *Journal of Human Resource and Sustainability Studies.* 4, 305-324. doi.org/10.4236/jhrss.2016.44030

Chen, H. M., and Chung, H. M. (2017). A scale for CEO personal brand measurement. *South African journal of business management*. 48(2), 23-32. doi.org/10.4102/sajbm.v48i2.25

Chen, Y. S., Raab, C., and Chen, C. C. (2017). The influence of celebrity chefs on restaurant customers’ behavior. *Journal of Hospitality Marketing & Management*. 26, 489-510. doi.org/10.1080/19368623.2017.1269305

Choi, S. M., and Rifon, N. J. (2012). It is a match: The impact of congruence between celebrity image and consumer ideal self on endorsement effectiveness. *Psychology & marketing*. 29, 639-650. doi.org/10.1002/mar.20550

Chu, F. Y., Dai, Y. X., Liu, J. Y., Chen, T. J., Chou, L. F., and Hwang, S. J. (2018). A Doctor’s Name as a Brand: A Nationwide Survey on Registered Clinic Names in Taiwan. *International journal of environmental research and public health*. 15, 1134. doi.org/10.3390/ijerph15061134

Clarke, T. B., Murphy, J., and Adler, J. (2016). Celebrity chef adoption and implementation of social media, particularly pinterest: A diffusion of innovations approach. *International Journal of Hospitality Management*. 57, 84-92. doi.org/10.1016/j.ijhm.2016.06.004

Click, M. A., Lee, H., and Holladay, H. W. (2013). Making monsters: Lady Gaga, fan identification, and social media. *Popular Music and Society*. 36, 360-379. doi.org/10.1080/03007766.2013.798546

Close, A. G., Moulard, J. G., and Monroe, K. B. (2011). Establishing human brands: determinants of placement success for first faculty positions in marketing. *Journal of the Academy of Marketing Science.* 39, 922-941. doi:10.1007/s11747-010-0221-6

Cocker, H. L., Banister, E. N., and Piacentini, M. G. (2015). Producing and consuming celebrity identity myths: unpacking the classed identities of Cheryl Cole and Katie Price. *Journal of Marketing Management.* 31, 502-524. doi:10.1080/0267257X.2015.1011196

Cole-Turner, R. (2019). Commodification and transfiguration: Socially mediated identity in technology and theology. *HTS Theological Studies*. 75(1), 1-11. doi.org/10.4102/hts.v75i1.5349

Coesemans, R., and De Cock, B. (2017). Self-reference by politicians on Twitter: Strategies to adapt to 140 characters. *Journal of Pragmatics*. 116, 37-50. doi.org/10.1016/j.pragma.2016.12.005

Colliander, J., Marder, B., Falkman, L. L., Madestam, J., Modig, E., and Sagfossen, S. (2017). The social media balancing act: Testing the use of a balanced self-presentation strategy for politicians using twitter. *Computers in Human Behavior*. 74, 277-285. doi.org/10.1016/j.chb.2017.04.042

Constantinescu, M. (2016). The Brand of an Athlete-Reconsidering Its Dimensions. *Journal of Emerging Trends in Marketing and Management*. 1, 354-363.

Corrigan, T. (2015). Media and Cultural Industries Internships: A Thematic Review and Digital Labor Parallels. *tripleC: Communication, Capitalism & Critique. Open Access Journal for a Global Sustainable Information Society*. 13, 336-350. doi.org/10.31269/triplec.v13i2.608

Cortsen, K. (2013). Annika Sörenstam–a hybrid personal sports brand. *Sport, Business and Management: An International Journal*. 3, 37-62. doi.org/10.1108/20426781311316898

Cottan-Nir, O. (2019). Toward a Conceptual Model for Determining CEO Brand Equity. *Corporate Reputation Review.* 22, 121-133. doi.org/10.1057/s41299-019-00063-3

Cottan-Nir, O., and Lehman-Wilzig, S. (2018). CEO Branding: Between Theory and Practice—Case Studies of Israeli Corporate Founders. *International Journal of Strategic Communication*. 12(2), 87-106. doi.org/10.1080/1553118x.2018.1425691

Craig, G. (2014). Kevin’s Predicaments: Power and Celebrity across the Political and Media Fields. *The International Journal of Press/Politics*. 19(1), 24-41. doi.org/10.1177/1940161213508813

Davies, F., and Slater, S. (2015). Unpacking celebrity brands through unpaid market communications. *Journal of Marketing Management.* 31, 665-684. doi.org/10.1080/0267257x.2014.1000941

Delisle, M. P., and Parmentier, M. A. (2016). Navigating person-branding in the fashion blogosphere. *Journal of Global Fashion Marketing.* 7, 211-224. doi.org/10.1080/20932685.2016.1167619

Desmarais, F. (2017). Who is the Athlete Endorser? A Cross-Cultural Exploration of Advertising Practitioners’ Views. *Journal of Global Marketing*. 30, 12-30. doi.org/10.1080/08911762.2016.1250976

Dionise, A. (2018). Communicating Royalty: A Study of Modern Monarchs’ Online Branding. *Elon Journal of Undergraduate Research in Communications*. 9, 18-26.

Draper, J., and McDonnell, A. M. (2018). Fashioning multiplatform masculinities: Gay personal style bloggers’ strategies of gendered self-representation across social media. *Men and Masculinities*. 21, 645-664. doi.org/10.1177/1097184x17696190

Duffy, B. (2015). Amateur, autonomous, and collaborative: Myths of aspiring female cultural producers in Web 2.0. *Critical Studies in Media Communication*. 32, 48-64. doi.org/10.1080/15295036.2014.997832

Duffy, B. E., and Hund, E. (2015). “Having it all” on social media: Entrepreneurial femininity and self-branding among fashion bloggers. *Social Media+ Society*. 1, 2056305115604337. doi.org/10.1177/2056305115604337

Duffy, B. E., and Pooley, J. D. (2017). “Facebook for academics”: the convergence of self-branding and social media logic on Academia. *Social Media+ Society*, *3*, 2056305117696523. doi.org/10.1177/2056305117696523

Duffy, B. E., and Pruchniewska, U. (2017). Gender and self-enterprise in the social media age: A digital double bind. *Information, Communication & Society*. 20, 843-859. doi.org/10.1080/1369118x.2017.1291703

Eagar, T., and Lindridge, A. (2015). Resolving contradictions in human brand celebrity and iconicity. *Consumer culture theory*. 17, 311-330. doi.org/10.1108/s0885-211120150000017015

Edmiston, D. (2014). Creating a personal competitive advantage by developing a professional online presence. *Marketing Education Review*. 24, 21-24. doi.org/10.2753/mer1052-8008240103

Ehrmann, T., Meiseberg, B., and Ritz, C. (2009). Superstar effects in deluxe gastronomy–An empirical analysis of value creation in German quality restaurants. *Kyklos*. 62, 526-541. doi.org/10.1111/j.1467-6435.2009.00449.x

Eke, H. N. (2012). Creating a digital footprint as a means of optimizing the personal branding of librarians in the digital society. *Webology*. 9, 31-40.

Ekhlasi, A., Talebi, K., and Alipour, S. (2015). Identifying the process of personal branding for entrepreneurs. *Asian Journal of Research in Marketing*. 4, 100-111.

Emmons, B., and Mocarski, R. (2014). She poses, he performs: A visual content analysis of male and female professional athlete Facebook profile photos. *Visual Communication Quarterly*. 21, 125-137. doi.org/10.1080/15551393.2014.893752

Erdoğmuş, N., and Esen, E. (2018). Constructing the CEO Personal Brand: The Case of Four Pioneering CEOs in Turkey. *Corporate Reputation Review*. 21, 37-49. doi.org/10.1057/s41299-017-0042-3

Erz, A., and Christensen, A. B. H. (2018). Transforming consumers into brands: Tracing transformation processes of the practice of blogging. *Journal of Interactive Marketing.* 43, 69-82. doi.org/10.1016/j.intmar.2017.12.002

Fetscherin, M. (2015). The CEO branding mix. *Journal of Business Strategy*. 36, 22-28. doi.org/10.1108/jbs-01-2015-0004

Fillis, I. R. (2015). The production and consumption activities relating to the celebrity artist. *Journal of Marketing Management.* 31, 646-664. doi:10.1080/0267257X.2014.988281

Finneman, T., Thomas, R. J., and Jenkins, J. (2019). “I Always Watched Eyewitness News Just to See Your Beautiful Smile”: Ethical Implications of US Women TV Anchors’ Personal Branding on Social Media. *Journal of Media Ethics*. 34(3), 146-159. doi.org/10.1080/23736992.2019.1638260

Flostrand, A., Ho, J. Y., and Krider, R. E. (2016). Implementing “Marketing Me” A Simulation Enhanced Variant for a Student Self-Marketing Exercise. *Journal of Marketing Education*. 38, 83-89. doi.org/10.1177/0273475316643567

Fournier, S. (2010). Taking stock in Martha Stewart: A cultural critique of the marketing practice of building person-brands. *ACR North American Advances*. 37-40.

Fresco, E. (2017). In LeBron James’ promotional skin: Self-branded athletes and fans’ immaterial labour. *Journal of Consumer Culture*, 1469540517745705. doi.org/10.1177/1469540517745705

Friel, T. J., and Duboff, R. S. (2009). The last act of a great CEO. *Harvard Business Review*. 87, 82-89.

Friend, A. (2016). The Edge of Ladyspace: Ladi6 and the Political Limits of Self-Branding. *MEDIANZ: Media Studies Journal of Aotearoa New Zealand*. 15, 1-24. doi.org/10.11157/medianz-vol15iss1id127

Gall, D. (2010). Librarian like a rock star: Using your personal brand to promote your services and reach distant users. *Journal of Library Administration.* 52, 549-558. doi:10.1080/01930826.2010.488928

Gandini, A. (2016). Digital work: Self-branding and social capital in the freelance knowledge economy. *Marketing Theory.* 16, 123-141. doi.org/10.1177/1470593115607942

García-Rapp, F., and Roc-Cuberes, C. (2017). Being an online celebrity: Norms and expectations of YouTube's beauty community. *First Monday*. 22(7). doi.org/10.5210/fm.v22i7.7788

Genz, S. (2015). My job is me: Postfeminist celebrity culture and the gendering of authenticity. *Feminist media studies*. 15(4), 545-561. doi.org/10.1080/14680777.2014.952758

Geurin, A. N. (2017). Elite female athletes’ perceptions of new media use relating to their careers: A qualitative analysis. *Journal of Sport Management*. 31, 345-359. doi.org/10.1123/jsm.2016-0157

Geurin-Eagleman, A. N., and Burch, L. M. (2016). Communicating via photographs: A gendered analysis of Olympic athletes’ visual self-presentation on Instagram. *Sport management review*. 19, 133-145. doi.org/10.1016/j.smr.2015.03.002

Green, M. R. (2016). The impact of social networks in the development of a personal sports brand. *Sports, Business and Management: An International Journal*. 6, 274-294. doi.org/10.1108/sbm-09-2015-0032

Gregory, K. (2019). Pushed and Pulled to the Internet: Self Employment in the Spiritual Marketplace. *American Behavioral Scientist*. 63, 208-224. doi.org/10.1177/0002764218794768

Hanusch, F. (2018). Political journalists’ corporate and personal identities on Twitter profile pages: A comparative analysis in four Westminster democracies. *New Media & Society*. 20, 1488-1505. doi.org/10.1177/1461444817698479

Hanusch, F., and Bruns, A. (2017). Journalistic branding on Twitter: A representative study of Australian journalists’ profile descriptions. *Digital journalism*. 5, 26-43. doi.org/10.1080/21670811.2016.1152161

Harrington, C. (2019). Neo-liberal subjectivity, self-branding and ‘my rape story’YouTube videos. *Critical Sociology*. 45, 1181-1194. doi.org/10.1177/0896920518778107

Harris, P., and Lock, A. (2001). Establishing the Charles Kennedy brand: a strategy for an election the result of which is a foregone conclusion. *Journal of Marketing Management*. 17, 943-956. doi.org/10.1362/026725701323366683

Harvey, A. (2018). The fame game: Working your way up the celebrity ladder in Kim Kardashian: Hollywood. *Games and Culture*. 13, 652-670. doi.org/10.1177/1555412018757872

Hasaan, A., Kerem, K., Biscaia, R., and Agyemang, K. J. (2016). Understanding the implications of athlete brand among fans. *Technics Technologies Education Management*. 11, 68-81.

Hasaan, S. A., Nawaz, S., Iqbal, S. J., and Khalid, J. (2018). Challenges That Make/Break the Athlete’s Quest to Become an Entrepreneur: A Qualitative Study About Fans’ Perceptions. *Physical Culture and Sport. Studies and Research*. 79, 53-61.

Hasaan, A., Biscaia, R., and Ross, S. (2019). Understanding athlete brand life cycle. *Sport in Society.* 1-42. doi.org/10.1080/17430437.2019.1624722

Hedman, U. (2015). J-Tweeters: Pointing towards a new set of professional practices and norms in journalism. *Digital Journalism*. 3, 279-297. doi.org/10.1080/21670811.2014.897833

Hedman, U., and Djerf-Pierre, M. (2013). The social journalist: Embracing the social media life or creating a new digital divide?. *Digital Journalism*. 1(3), 368-385. doi.org/10.1080/21670811.2013.776804

Hendrawan, A., and Nahdiah, S. (2019). Personal branding analysis of food blogger cindy lulaby through instagram social media. *International Journal of Scientific and Technology Research*. 8, 164-168.

Henning, S., and Padayachee, S. (2018). A Conceptual Framework for the Management of a Personal Leadership Brand within the Tourism and Hospitality Industry. *African Journal of Hospitality, Tourism and Leisure*. 7, 1-26.

Hearn, A. (2016). Trump’s “reality” hustle. *Television & New Media*. 17, 656-659. doi.org/10.1177/1527476416652699

Hernando, E., and Campo, S. (2017). Does the artist's name influence the perceived value of an art work?. *International Journal of Arts Management*. 19, 46.

Hodge, C., and Walker, M. (2015). Personal branding: a perspective from the professional athlete-level-of-analysis. *International Journal of Sport Management and Marketing*. 16, 112-131. doi.org/10.1504/ijsmm.2015.074920

Holmberg, I., and Strannegård, L. (2015). Students’ self-branding in a Swedish business school. *International Studies of Management & Organization*. 45, 180-192. doi.org/10.1080/00208825.2015.1006017

Holton, A. E., and Molyneux, L. (2017). Identity lost? The personal impact of brand journalism. *Journalism*. 18, 195-210. doi.org/10.1177/1464884915608816

Hood, K. M., Robles, M., and Hopkins, C. D. (2014). Personal branding and social media for students in today's competitive job market. *The journal of research in business education*. 56, 33.

Hotez, P. J. (2018). Crafting your scientist brand. *PLoS biology*. 16, e3000024. doi.org/10.1371/journal.pbio.3000024

Huang, S. C. T., and Huang, T. J. (2016). The evolution of fan kingdom: the rising, expansion, and challenges of human brands. *Asia Pacific Journal of Marketing and Logistics*. 28, 683-708. doi.org/10.1108/apjml-07-2015-0111

Humphrey Jr, W., Laverie, D., and Shields, A. (2019). Exploring the Effects of Encouraging Student Performance With Text Assignment Reminders. *Journal of Marketing Education*, 0273475319836271. doi.org/10.1177/0273475319836271

Ilieş, V. I. (2018). Strategic Personal Branding for Students and Young Professionals. *Cross-Cultural Management Journal*. 20, 43-51.

Ilina, I., Kryukova, E., Potekhina, E., Abyzova, E., and Shadskaja, I. (2017). Russian lectures at the crossroads of reforms: strategies of survival and adaptation. *European Research Studies Journal*. 20, 86-97. doi.org/10.35808/ersj/667

Ioan, C. A., Luca, F., and Sasu, C. (2014). Personal Marketing of Doctors in Context of Social Networks. *Cross-Cultural Management Journal*. 6, 369-376.

Jillapalli, R. K., and Jillapalli, R. (2014). Do professors have customer-based brand equity?. *Journal of Marketing for Higher Education*. 24, 22-40. doi.org/10.1080/08841241.2014.909556

Jillapalli, R. K., and Wilcox, J. B. (2010). Professor brand advocacy: do brand relationships matter?. *Journal of Marketing Education*. 32, 328-340. doi.org/10.1177/0273475310380880

Johns, R., and English, R. (2016). Transition of self: Repositioning the celebrity brand through social media—The case of Elizabeth Gilbert. *Journal of Business Research*. 69, 65-72. doi.org/10.1016/j.jbusres.2015.07.021

Johnson, K. M. (2015). Non-technical skills for IT professionals in the landscape of Social Media. *American Journal of Business and Management*. 4, 102-122. doi.org/10.11634/216796061504668

Johnson, K. (2017). The importance of personal branding in social media: educating students to create and manage their personal brand. *International Journal of Education and Social Science*. 4, 21-27.

Jones, B. (2010). Climbing the greasy pole: Promotion in British politics. *The Political Quarterly*. 81, 616-626. doi.org/10.1111/j.1467-923x.2010.02132.x

Jones, B., and Leverenz, C. (2017). Building Personal Brands with Digital Storytelling ePortfolios. *International Journal of ePortfolio*. 7, 67-91.

Jukes, S. (2019). Crossing the line between news and the business of news: Exploring journalists’ use of Twitter. *Media and Communication.* 7, 248-258. doi.org/10.17645/mac.v7i1.1772

Kahanov, L., and Andrews, L. (2001). A survey of athletic training employers' hiring criteria. *Journal of athletic training*. 36, 408-412.

Kakitek, A. (2018). Application of Aaker’s Brand Personality Scale on Human Brands in Surf Sports. *Journal of Management and Business Administration. Central Europe*. 26, 11-31. doi.org/10.7206/jmba.ce.2450-7814.240

Kalbande, D. T. (2019). Digital footprint for the personal branding of librarians in the digital society. *Library Philosophy and Practice*. 1-11.

Kalia, V., Patel, A. K., Moriarity, A. K., and Canon, C. L. (2017). Personal branding: a primer for radiology trainees and radiologists. *Journal of the American College of Radiology*. 14, 971-975. doi.org/10.1016/j.jacr.2017.03.017

Kaneva, N., and Klemmer, A. (2016). The rise of brandidates? A cultural perspective on political candidate brands in postmodern consumer democracies. *Journal of Customer Behaviour*. 15, 299-313. doi.org/10.1362/147539216x14594362874054

Karaduman, I. (2013). The effect of social media on personal branding efforts of top level executives. *Procedia-social and behavioral sciences*. 99, 465-473. doi.org/10.1016/j.sbspro.2013.10.515

Keller, J. M. (2014). Fiercely Real?: Tyra Banks and the making of new media celebrity. *Feminist Media Studies*. 14, 147-164. doi.org/10.1080/14680777.2012.740490

Kelting, K., and Rice, D. H. (2013). Should we hire David Beckham to endorse our brand? Contextual interference and consumer memory for brands in a celebrity's endorsement portfolio. *Psychology & Marketing*. 30, 602-613. doi.org/10.1002/mar.20631

Kerrigan, F., Brownlie, D., Hewer, P., and Daza-LeTouze, C. (2011). ‘Spinning’ Warhol: Celebrity brand theoretics and the logic of the celebrity brand. *Journal of Marketing Management.* 27, 1504-1524. doi.org/10.1080/0267257x.2011.624536

Khamis, S., Ang, L., and Welling, R. (2017). Self-branding, ‘micro-celebrity’ and the rise of Social Media Influencers. *Celebrity Studies*. 8, 191-208. doi.org/10.1080/19392397.2016.1218292

Kretz, G., and de Valck, K. (2010). ‘Pixelize me!’: digital storytelling and the creation of archetypal myths through explicit and implicit self-brand association in fashion and luxury blogs. *Research in consumer behavior*. 12, 313-329. doi.org/10.1108/s0885-2111(2010)0000012015

Kristiansen, E., and Williams, A. S. (2015). Communicating the athlete as a brand: An examination of LPGA star Suzann Pettersen. *International Journal of Sport Communication*. 8, 371-388. doi.org/10.4135/9781526438201

Kucharska, W., and Mikołajczak, P. (2018). Personal branding of artists and art-designers: necessity or desire?. *Journal of Product & Brand Management*. 27, 249-261. doi.org/10.1108/jpbm-01-2017-1391

Kunkel, T., Walker, M., and Hodge, C. M. (2019). The influence of advertising appeals on consumer perceptions of athlete endorser brand image. *European Sport Management Quarterly*. 19, 373-395. doi.org/10.1080/16184742.2018.1530688

Lebel, K., and Danylchuk, K. (2014). An Audience Interpretation of Professional Athlete Self-Presentation on Twitter. *Journal of Applied Sport Management*. 6.

Lee, J. W., and Cavanaugh, T. (2016). Building your brand: The integration of infographic resume as student self-analysis tools and self-branding resources. *Journal of Hospitality, Leisure, Sport & Tourism Education*. 18, 61-68. doi.org/10.1016/j.jhlste.2016.03.001

Levin, E., Rixon, A., and Keating, M. (2019). How can a 'Sense of Belonging' inform your teaching strategy? Reflections from a core Business unit. A Practice Report. *Student Success*. 10, 71-79. doi.org/10.5204/ssj.v10i2.1307

Lindridge, A., and Eagar, T. (2015). ’And Ziggy played guitar': Bowie, the market, and the emancipation and resurrection of Ziggy Stardust. *Journal of Marketing Management.* 31, 546-576. doi:10.1080/0267257X.2015.1014395

Little, B. (2012). Identifying key trends in sales–from a training perspective. *Industrial and Commercial Training*. 44, 103-108. doi.org/10.1108/00197851211202948

Liu, R., and Suh, A. (2017). Self-branding on social media: An analysis of style bloggers on Instagram. *Procedia Computer Science*. 124, 12-20. doi.org/10.1016/j.procs.2017.12.124

Lobpries, J., Bennett, G., and Brison, N. (2017). Mary Ann to her Ginger: comparing the extended brand identity of two elite female athletes. *International Journal of Sports Marketing and Sponsorship*. 18, 347-362. doi.org/10.1108/ijsms-05-2016-0028

Lobpries, J., Bennett, G., and Brison, N. (2018). How I Perform is Not Enough: Exploring Branding Barriers Faced by Elite Female Athletes. *Sport Marketing Quarterly*. 27, 5-17.

Loroz, P. S., and Braig, B. M. (2015). Consumer attachments to human brands: The “Oprah Effect”. *Psychology & Marketing.* 32, 751-763. doi:10.1002/mar.20815

Lovelock, M. (2017). ‘Is every YouTuber going to make a coming out video eventually?’: YouTube celebrity video bloggers and lesbian and gay identity. *Celebrity Studies.* 8, 87-103. doi.org/10.1080/19392397.2016.1214608

Luca, F.-A., Ioan, C. A., and Sasu, C. (2015). The Importance of the Professional Personal Brand. The Doctors’ Personal Brand. *Procedia Economics and Finance.* 20, 350-357. doi:10.1016/S2212-5671(15)00083-0.

Maguire, E. (2015). Self-branding, hotness, and girlhood in the video blogs of Jenna Marbles. *Biography - An Interdisciplinary Quarterly.* 38, 72-86. doi.org/10.1353/bio.2015.0006

Maiksteniene, K. (2009). Modeling Brand Alliance Effects in Professional Services. *World Academy of Science, Engineering and Technology*. 3, 1378-1386.

Makkai, J. A. (2016). Personal branding of contemporary novelists in the digital age. *Journal of Media Research-Revista de Studii Media*. 9, 100-105.

Manai, A., and Holmlund, M. (2015). Self-marketing brand skills for business students. *Marketing Intelligence and Planning.* 33, 749-762. doi:10.1108/MIP-09-2013-0141

Manurung, A. D. R. (2015). The Influence of Heroic Leadership and Learning Organization to Work Achievement with Authentic Personal Branding as Mediator. *Mediterranean Journal of Social Sciences*. 6, 18-25. doi.org/10.5901/mjss.2015.v6n5s5p18

Marcus, S. (2015). Celebrity 2.0: the case of Marina Abramović. *Public culture*. 27, 21-52. doi.org/10.1215/08992363-2798331

Matenge, T. M. (2013). Botswana music: Is personal branding the missing factor for growth?. *Global Advanced Research Journal of Arts and Humanities*. 2, 48-53.

Mathys, J., Burmester, A. B., and Clement, M. (2016). What drives the market popularity of celebrities? A longitudinal analysis of consumer interest in film stars. *International Journal of Research in Marketing*. 33, 428-448. doi.org/10.1016/j.ijresmar.2015.09.003

Marwitz, K. K., Hertig, J. B., and Weber, R. J. (2018). One Chance for Your Best First Impression: Tips for New Pharmacists. *Hospital pharmacy*. 53, 148-151. doi.org/10.1177/0018578718766096

McGhee, T. (2012). The rise and rise of athlete brand endorsements. *Journal of Brand Strategy*. 1, 79-84.

McCorkle, D. E., Alexander, J. F., and Diriker, M. F. (1992). Developing self-marketing skills for student career success. *Journal of Marketing Education*. 14, 57-67. doi.org/10.1177/027347539201400108

McCorkle, D. E., Alexander, J. F., Reardon, J., and Kling, N. D. (2003). Developing self-marketing skills: Are marketing students prepared for the job search?. *Journal of Marketing Education*. 25, 196-207. doi.org/10.1177/0273475303257517

McFadden, S. E. (2018). They’re good blogs, Brent:(Mostly) painless branding for information professionals. *Public Library Quarterly*. 37, 306-317. doi.org/10.1080/01616846.2018.1513259

McKernan, B. (2011). Politics and celebrity: A sociological understanding. *Sociology Compass*. 5, 190-202. doi.org/10.1111/j.1751-9020.2011.00359.x

Medveschi, I., and Frunza, S. (2018). Political brand, symbolic construction and public image communication. *Journal for the Study of Religions and Ideologies*. 17, 137-152.

Meiseberg, B. (2014). Trust the artist versus trust the tale: performance implications of talent and self-marketing in folk music. *Journal of Cultural Economics*. 38, 9-42. doi.org/10.1007/s10824-012-9196-0

Milewicz, C. M., and Milewicz, M. C. (2014). The branding of candidates and parties: The US news media and the legitimization of a new political term. *Journal of Political Marketing*. 13, 233-263. doi.org/10.1080/15377857.2014.958364

Mills, S., Patterson, A., and Quinn, L. (2015). Fabricating celebrity brands via scandalous narrative: crafting, capering and commodifying the comedian, Russell Brand. *Journal of Marketing Management.* 31, 599-615. doi:10.1080/0267257X.2015.1005116

Mishra, S. (2019). When patients connect with physicians on facebook: physician perspectives on benefits, challenges, and strategies for managing interaction. *Health and Technology*. 9, 505-515. doi.org/10.1007/s12553-018-0273-z

Molyneux, L. (2015). What journalists retweet: Opinion, humor, and brand development on Twitter. *Journalism*. 16, 920-935. doi.org/10.1177/1464884914550135

Molyneux, L. (2019). A Personalized Self-image: Gender and Branding Practices Among Journalists. *Social Media+ Society*. 5, 2056305119872950. doi.org/10.1177/2056305119872950

Molyneux, L., and Holton, A. (2015). Branding (health) journalism: Perceptions, practices, and emerging norms. *Digital journalism*. 3, 225-242. doi.org/10.1080/21670811.2014.906927

Molyneux, L., Holton, A., and Lewis, S. C. (2018). How journalists engage in branding on Twitter: Individual, organizational, and institutional levels. *Information, Communication & Society*. 21, 1386-1401. doi.org/10.1080/1369118x.2017.1314532

Molyneux, L., Lewis, S. C., and Holton, A. E. (2019). Media work, identity, and the motivations that shape branding practices among journalists: An explanatory framework. *New media & society*. 21, 836-855. doi.org/10.1177/1461444818809392

Morais, D. G. (2013). Branding Iron: Eugen Sandow's “Modern” Marketing Strategies, 1887-1925. *Journal of Sport History*. 40, 193-214.

Moulard, J. G., Rice, D. H., Garrity, C. P., and Mangus, S. M. (2014). Artist Authenticity: How Artists' Passion and Commitment Shape Consumers' Perceptions and Behavioral Intentions across Genders. *Psychology and Marketing.* 31, 576-590. doi:10.1002/mar.20719

Mudambi, S. M., Sinha, J. I., and Taylor, D. S. (2019). Why B-to-B CEOs should be more social on social media. *Journal of Business-to-Business Marketing*. 26, 103-105. doi.org/10.1080/1051712x.2019.1565144

Munden, R. F. (2015). Marketing and branding for a radiologist. *Journal of the American College of Radiology*. 12, 130-131. doi.org/10.1016/j.jacr.2014.10.015

Muñiz Jr, A. M., Norris, T., and Fine, G. A. (2014). Marketing artistic careers: Pablo Picasso as brand manager. *European Journal of Marketing*. 48, 68-88. doi.org/10.1108/ejm-01-2011-0019

Murphy, P. (2010). The intractability of reputation: Media coverage as a complex system in the case of Martha Stewart. *Journal of Public Relations Research*. 22, 209-237. doi.org/10.1080/10627261003601648

Myers, J. (2017). Brand yourself on youtube: The design, execution, and reflection of a three-fold experiential exercise. *Journal of Marketing Development and Competitiveness*. 11.

Nayar, K. I. (2015). You Did (n't) Build That: Audience Reception of a Reality Television Star's Transformation from a Real Housewife to a Real Brand. *The Journal of Popular Culture*. 48, 3-16. doi.org/10.1111/jpcu.12229

Nolan, L. (2015). The impact of executive personal branding on non-profit perception and communications. *Public Relations Review*. 41, 288-292. doi.org/10.1016/j.pubrev.2014.11.001

Ogutu, R. P. and Ougo, R. T. (2016). The relationship between personal branding and career success: a case of employees at geothermal development company in kenya. International Journal of Economics, Commerce and Management. 4, 282-306.

Olausson, U. (2017). The reinvented journalist: The discursive construction of professional identity on Twitter. *Digital Journalism*. 5, 61-81. doi.org/10.1080/21670811.2016.1146082

Olausson, U. (2018). The celebrified journalist: Journalistic self-promotion and branding in celebrity constructions on Twitter. *Journalism Studies*. 19, 2379-2399. doi.org/10.1080/1461670x.2017.1349548

O'Reilly, N. J., and Braedley, L. A. (2008). Celebrity athletes and athletic clothing design: branding female tennis players. *International Journal of Sport Management and Marketing*. 3, 119-139. doi.org/10.1504/ijsmm.2008.015964

Otnes, C. C., and Maclaran, P. (2018). Royalty: marketplace icons. *Consumption Markets & Culture.* 21, 65-75. doi.org/10.1080/10253866.2016.1220371

Ottovordemgentschenfelde, S. (2017). ‘Organizational, professional, personal’: An exploratory study of political journalists and their hybrid brand on Twitter. *Journalism*. 18, 64-80. doi.org/10.1177/1464884916657524

Pagis, M., and Ailon, G. (2017). The paradoxes of self-branding: An analysis of consultants’ professional web pages. *Work and Occupations*. 44, 243-267. doi.org/10.1177/0730888417709327

Pahwa, S. (2019). Mediating modesty on Saudi YouTube: from postfeminist to posthuman performance. *Women & Performance: a journal of feminist theory*. 29, 162-178. doi.org/10.1080/0740770x.2019.1621602

Parker, B. T. (2012). Candidate brand equity valuation: A comparison of US presidential candidates during the 2008 primary election campaign. *Journal of Political Marketing*. 11, 208-230. doi.org/10.1080/15377857.2012.699424

Parmentier, M. A., and Fischer, E. (2012). How athletes build their brands. *International Journal of Sport Management and Marketing.* 11, 106-124. doi:10.1504/IJSMM.2012.045491

Parmentier, M. A., Fischer, E., and Reuber, A. R. (2013). Positioning person brands in established organizational fields. *Journal of the Academy of Marketing Science.* 41, 373-387. doi:10.1007/s11747-012-0309-2

Parris, D. L., Troilo, M. L., Bouchet, A., and Peachey, J. W. (2014). Action sports athletes as entrepreneurs: Female professional wakeboarders, sponsorship, and branding. *Sport Management Review*. 17, 530-545. doi.org/10.1016/j.smr.2013.12.005

Pérez-Curiel, C., and Limón-Naharro, P. (2019). Political influencers. A study of Donald Trump’s personal brand on Twitter and its impact on the media and users. *Communication and Society*. 32, 57-75. doi.org/10.15581/003.32.1.57-76

Persis Murray, D. (2015). “This Is What Was Birthed” Motherhood and Postfeminist Self-Branding in Tyra Banks’ Brand. *Journal of Communication Inquiry*. 39, 232-248. doi.org/10.1177/0196859915576373

Pick, D., Weber, P., Connell, J., Geneste, L. A., and de Klerk, S. (2015). The creative industries: an entrepreneurial bricolage perspective. *Management Decision*. 53, 828-842. doi.org/10.1108/md-03-2014-0169

Pihl, C. (2013). In the borderland between personal and corporate brands – the case of professional bloggers. *Journal of Global Fashion Marketing.* 4, 112-127. doi.org/10.1080/20932685.2013.763474

Preece, C. (2015). The authentic celebrity brand: unpacking Ai Weiwei’s celebritised selves. *Journal of Marketing Management*. 31, 616-645. doi.org/10.1080/0267257x.2014.1000362

Preece, C., and Kerrigan, F. (2015). Multi-stakeholder brand narratives: an analysis of the construction of artistic brands. *Journal of Marketing Management.* 31, 1207-1230. doi:10.1080/0267257X.2014.997272

Price, J., Farrington, N., and Hall, L. (2013). Changing the game? The impact of Twitter on relationships between football clubs, supporters and the sports media. *Soccer & Society*. 14, 446-461. doi.org/10.1080/14660970.2013.810431

Pruchniewska, U. M. (2018). Branding the self as an “authentic feminist”: negotiating feminist values in post-feminist digital cultural production. *Feminist Media Studies*. 18, 810-824. doi.org/10.1080/14680777.2017.1355330

Radford, M. L., Kitzie, V., Mikitish, S., Floegel, D., Radford, G. P., and Connaway, L. S. (2018). Investigating practices for building an ethical and sustainable scholarly identity with online platforms and social networking sites. *Proceedings of the Association for Information Science and Technology*. 55, 404-413. doi.org/10.1002/pra2.2018.14505501044

Raftari, M., and Amiri, B. (2014). An entrepreneurial business model for personal branding: proposing a framework. *Journal of Entrepreneurship, Business and Economics*. 2, 121-139.

Rascher, D., Eddy, T., and Hyun, G. (2017). What Drives Endorsement Earnings for Superstar Athletes? *Journal of Applied Sport Management*. 9. doi.org/10.18666/JASM-2017-V9-I2-7997

Razeghi, Y., Roosta, A., Gharache, M., and Alemtabriz, A. (2016). Understanding the role of entrepreneur's personal brand in SMEs total brand. *International Business and Management*. 12, 47-57.

Reif-Lehrer, L. (1992). Self-marketing ensures that good scientists get credit they deserve. *Scientist*. 6.

Resnick, S. M., Cheng, R., Simpson, M., and Lourenço, F. (2016). Marketing in SMEs: a “4Ps” self-branding model. *International Journal of Entrepreneurial Behavior & Research*. 22, 155-174. doi.org/10.1108/ijebr-07-2014-0139

Robson, K. (2019). Motivating professional student behavior through a gamified personal branding assignment. *Journal of Marketing Education*. 41, 154-164. doi.org/10.1177/0273475318823847

Rozanova, N. N. (2017). Priority value characteristics of the governor’s personal brand (on the example of the Smolensk Region). *RUDN Journal of Sociology*. 17, 542-554. doi.org/10.22363/2313-2272-2017-17-4-542-554

Saboo, A. R., Kumar, V., and Ramani, G. (2016). Evaluating the impact of social media activities on human brand sales. *International Journal of Research in Marketing*. 33, 524-541. doi.org/10.1016/j.ijresmar.2015.02.007

Safitri, Y. (2017). Personal branding through fashion blogging. *Humaniora*. 8, 69-78. doi.org/10.21512/humaniora.v8i1.3697

Sassenberg, A. M., Verreynne, M. L., and Johnson Morgan, M. (2012). A sport celebrity brand image: a conceptual model. *International Journal of Organisational Behaviour*. 17, 108-121.

Scammell, M. (2007). Political brands and consumer citizens: The rebranding of Tony Blair. *The Annals of the American Academy of Political and Social Science*. 611, 176-192. doi.org/10.1177/0002716206299149

Scharff, C. (2015). Blowing your own trumpet: Exploring the gendered dynamics of self‐promotion in the classical music profession. *The Sociological Review*. 63, 97-112. doi.org/10.1111/1467-954x.12243

Scheidt, S., Gelhard, C., Strotzer, J., and Henseler, J. (2018). In for a penny, in for a pound? Exploring mutual endorsement effects between celebrity CEOs and corporate brands. *Journal of Product & Brand Management.* 27, 203-220. doi.org/10.1108/jpbm-07-2016-1265

Schlosser, F., McPhee, D. M., and Forsyth, J. (2017). Chance events and executive career rebranding: implications for career coaches and nonprofit HRM. *Human Resource Management*. 56, 571-591. doi.org/10.1002/hrm.21789

Schneider, K. G. (2012). Personal Branding for Librarians: Distinguishing yourself from the professional herd. *American Libraries*. 43, 34-37.

Schroeder, J. E. (2005). The artist and the brand. *European Journal of Marketing.* 39, 1291-1305. doi:10.1108/03090560510623262

Schultz, B., and Sheffer, M. L. (2012). Personal branding still in future for most newspaper reporters. *Newspaper Research Journal*. 33, 63-77. doi.org/10.1177/073953291203300406

Schwartz, D., and Vogan, T. (2017). The Players' Tribune: Self-Branding and Boundary Work in Digital Sports Media. *Journal of Sports Media*. 12, 45-63. doi.org/10.1353/jsm.2017.0002

Shafaei, A., Nejati, M., and Maadad, N. (2019). Brand equity of academics: demystifying the process. *Journal of Marketing for Higher Education*. 29, 121-133. doi.org/10.1080/08841241.2019.1605438

Sheikh, A., and Lim, M. (2011). Engineering consultants' perceptions of corporate branding: A case study of an international engineering consultancy. *Industrial Marketing Management.* 40, 1123-1132. doi:10.1016/j.indmarman.2011.09.006

Shek, D. T., Chung, P. P., and Leung, H. (2015). Manufacturing economy vs. service economy: implications for service leadership. *International Journal on Disability and Human Development*. 14, 205-215. doi.org/10.1515/ijdhd-2015-0402

Shuart, J. (2007). Heroes in sport: assessing celebrity endorser effectiveness. *International Journal of Sports Marketing and Sponsorship*. 8, 11-25. doi.org/10.1108/ijsms-08-02-2007-b004

Shuker, L. E. (2014). 'It'll look good on your personal statement': Self-marketing amongst university applicants in the United Kingdom. *British Journal of Sociology of Education.* 35, 224-243. doi:10.1080/01425692.2012.740804

Smith, D. (2014). Charlie is so ‘English’-like: Nationality and the branded celebrity person in the age of YouTube. *Celebrity studies*. 5, 256-274. doi.org/10.1080/19392397.2014.903160

Speed, R., Butler, P., and Collins, N. (2015). Human Branding in Political Marketing: Applying Contemporary Branding Thought to Political Parties and Their Leaders. *Journal of Political Marketing.* 14, 129-151. doi:10.1080/15377857.2014.990833

Stanton, A. D. A., and Stanton, W. W. (2013). Building" Brand Me": Creating a personal brand statement. *Marketing Education Review*. 23, 81-86. doi.org/10.2753/mer1052-8008230113

Staskeviciute-Butiene, I., Bradauskiene, K., and Crespo-Hervas, J. (2014). Athletes' personal brand as a success factor for start-up. *Transformations in Business & Economics*. 13, 525-540.

Street, J. (2004). Celebrity politicians: Popular culture and political representation. *The British journal of politics and international relations*. 6, 435-452. doi.org/10.1111/j.1467-856x.2004.00149.x

Summers, J., and Morgan, M. J. (2008). More than just the media: Considering the role of public relations in the creation of sporting celebrity and the management of fan expectations. *Public Relations Review*. 34, 176-182. doi.org/10.1016/j.pubrev.2008.03.014

Swan, A. A. (2016). The iconic body: Mussolini unclothed. *Modern Italy*. 21, 361-381. doi.org/10.1017/mit.2016.54

Sweeney, M., Docherty-Hughes, J., and Lynch, P. (2018). Lifestyling entrepreneurs’ sociological expressionism. *Annals of Tourism Research*. 69, 90-100. doi.org/10.1016/j.annals.2018.01.006

Takács, I., Takács, V., and Kondor, A. (2018). Empirical Investigation of Chief Executive Officers' Personal Brand. *Periodica Polytechnica Social and Management Sciences*. 26, 112-120. doi.org/10.3311/ppso.10883

Tanner, R. J., and Maeng, A. (2012). A tiger and a president: Imperceptible celebrity facial cues influence trust and preference. *Journal of Consumer Research*. 39, 769-783. doi.org/10.1086/665412

Tarnovskaya, V. (2017). Reinventing personal branding building a personal brand through content on YouTube. *Journal of International Business Research and Marketing.* 3, 29-35. doi.org/10.18775/jibrm.1849-8558.2015.31.3005

Taylor, K. A. (2003). Marketing yourself in the competitive job market: An innovative course preparing undergraduates for marketing careers. *Journal of Marketing Education*. 25, 97-107. doi.org/10.1177/0273475302250577

Thomas, L. C. (2011). Building blocks for personal brands. *Journal of Web Librarianship.* 5, 142-145. doi:10.1080/19322909.2011.572439

Thompson-Whiteside, H., Turnbull, S., and Howe-Walsh, L. (2018). Developing an authentic personal brand using impression management behaviours. *Qualitative Market Research: An International Journal*. 21, 166-181. doi.org/10.1108/qmr-01-2017-0007

Treadway, D. C., Adams, G. L., Ranft, A. L., and Ferris, G. R. (2009). A meso-level conceptualization of CEO celebrity effectiveness. *The Leadership Quarterly*. 20, 554-570. doi.org/10.1016/j.leaqua.2009.04.008

Trepanier, S., and Gooch, P. (2014). Personal branding and nurse leader professional image. *Nurse Leader.* 12, 51-53. doi:10.1016/j.mnl.2014.03.005

Tripp, C., Jensen, T. D., and Carlson, L. (1994). The effects of multiple product endorsements by celebrities on consumers' attitudes and intentions. *Journal of consumer research*. 20, 535-547. doi.org/10.1086/209368

Tsiotsou, R. H., Wirtz, J., Mitsis, A., and Leckie, C. (2016). Validating and extending the sport brand personality scale. *Journal of Service Theory and Practice*. 26, 203-221. doi.org/10.1108/jstp-08-2014-0185

Turri, A. M., Smith, K. H., and Kemp, E. (2013). Developing affective brand commitment through social media. *Journal of Electronic Commerce Research*. 14, 201-214.

Tussyadiah, I. P., and Park, S. (2018). When guests trust hosts for their words: Host description and trust in sharing economy. *Tourism Management*. 67, 261-272. doi.org/10.1016/j.tourman.2018.02.002

Uymaz, A. O. (2016). The Influence of Transformational Leadership on Personal Branding through the Learning Organization and Consideration of Future Consequences. *International Journal of Academic Research in Business and Social Sciences*. 6, 2222-6990. doi.org/10.6007/ijarbss/v6-i3/2028

Van Hove, F., Asdourian, B., and Bourgeois, D. (2018). My tweets are (not) my own! “Normalizing” journalists’ branding and digital identity on Twitter. *Popular Communication*. 16, 263-275. doi.org/10.1080/15405702.2018.1535659

Van Nuenen, T. (2016). Here I am: Authenticity and self-branding on travel blogs. *Tourist Studies*. 16, 192-212. doi.org/10.1177/1468797615594748

Van Steenburg, E., and Guzmán, F. (2019). The influence of political candidate brands during the 2012 and 2016 US presidential elections. *European Journal of Marketing*. 53, 2629-2656. doi.org/10.1108/ejm-06-2018-0399

Wade, J. B., Porac, J. F., Pollock, T. G., and Graffin, S. D. (2006). The burden of celebrity: The impact of CEO certification contests on CEO pay and performance. *Academy of Management Journal*. 49, 643-660. doi.org/10.5465/amj.2006.22083021

Walsh, P., and Williams, A. (2017). To extend or not extend a human brand: An analysis of perceived fit and attitudes toward athlete brand extensions. *Journal of Sport Management*. 31, 44-60. doi.org/10.1123/jsm.2015-0314

Wang, S. J., Hsu, C. P., Huang, H. C., and Chen, C. L. (2015). How readers’ perceived self-congruity and functional congruity affect bloggers’ informational influence. *Online Information Review*. 39, 537-555. doi.org/10.1108/oir-02-2015-0063

Watson, M. (2019). Using Professional Online Portfolios to Enhance Student Transition Into the Poststudent World. *Business and Professional Communication Quarterly*. 82, 153-168. doi.org/10.1177/2329490618824703

Weikop, C. (2012). Ernst Ludwig Kirchner as his Own Critic: The Artist's Statements as Stratagems of Self-Promotion. *Forum for Modern Language Studies*. 48, 406-420. doi.org/10.1093/fmls/cqs031

Williams, A. S., Kim, D. Y., Choi, W., and Walsh, P. (2015). What Children Love About Athletes: An Exploratory Assessment of Athlete Brand Associations Among Youth Consumers. *Global Sport Business Journal*. 3, 63-77.

Wilson, J. A., and Liu, J. (2012). From laconophilia to ‘The Sportan’: balancing athletic excellence, sponsorship, branding and career prospects. *International Journal of Sport Management and Marketing*. 11, 125-142. doi.org/10.1504/ijsmm.2012.045492

Wohlfeil, M., and Whelan, S. (2012). “Saved!” by Jena Malone: An introspective study of a consumer's fan relationship with a film actress. *Journal of Business Research*. 65, 511-519. doi.org/10.1016/j.jbusres.2011.02.030

Zamudio, C., Wang, Y., and Haruvy, E. E. (2013). Human brands and mutual choices: an investigation of the marketing assistant professor job market. *Journal of the Academy of Marketing Science.* 41, 722-736. doi:10.1007/s11747-013-0341-x

Zamudio, C., and Meng, M. (2015). Which Modeling Scholars Get Promoted, and How Fast?. *Customer Needs and Solutions*. 2, 91-104. doi.org/10.1007/s40547-014-0030-z

Zeng, Y., and Song, Y. (2018). The social foreign correspondent: reconfiguring journalistic branding research in the age of social media. *Popular Communication*. 16, 293-308. doi.org/10.1080/15405702.2018.1543888

Zhou, Y., and Tainsky, S. (2017). Enhanced Brand Credibility of American Athletes with International Teammates. *Sport Marketing Quarterly*. 26, 63-74.
